# Divergent philosophical commitments in neuroscience: Evidence from a global survey

**DOI:** 10.1073/pnas.2610776123

**Published:** 2026-07-15

**Authors:** Fabián Navarro-Peña, Gonzalo Arrondo, Nathaniel F. Barrett, Francisco Güell, Gabriel Madirolas, José Ignacio Murillo, Javier Sánchez-Cañizares, Javier Bernacer

**Affiliations:** ^a^ Mind-Brain Group, Institute for Culture and Society, University of Navarra, Pamplona 31006, Spain; ^b^ Research Center on Animal Cognition, Center for Integrative Biology, CNRS, Toulouse University, Toulouse 31062, France; ^c^International Center for Neuroscience and Ethics, Tatiana Foundation, Madrid 28010, Spain

**Keywords:** free will, interdisciplinarity, mind–brain problem, neuroessentialism

## Abstract

The mind–brain relationship is a foundational yet underexplored dimension of contemporary neuroscience, shaping research framing, data interpretation, and public communication. We conducted a large-scale international survey of 2,657 neuroscientists to assess their views on the mind–brain problem, free will, and the future of the discipline. Our results reveal a complex and sometimes paradoxical worldview. While 64% of participants endorse reductive physicalism—the view that mental activity is fully reducible to brain function—only 17.5% reject free will. Despite estimating that current knowledge covers only a limited portion of brain structure and function (30%), respondents express marked optimism about the future of neuroscience, including the prospect of fully understanding the human being and mental disorders through neuroscience, and even achieving mind reading and mental uploading. A principal component analysis identified five latent dimensions, some of which vary systematically across sociodemographic and disciplinary factors. These findings indicate that neuroscientists hold diverse philosophical commitments that diverge from common narratives of hard determinism. The prevalence of neuroessentialist views further highlights the need for sustained interdisciplinary dialogue to address the conceptual, ethical, and societal implications of neuroscientific progress.

## Significance

This study presents a large-scale international survey of the philosophical worldviews of 2,657 neuroscientists. We reveal a complex landscape in which 64% endorse reductive physicalism, yet only 17.5% reject free will. Despite widespread acknowledgment of limited current understanding of the brain, respondents display marked neurooptimism regarding the future capacity of neuroscience to explain human nature and mental disorders. Attitudes vary systematically with sociodemographic and disciplinary background. These findings matter because scientists’ foundational beliefs shape research directions, data interpretation, and public discourse. Our results highlight the need for sustained interdisciplinary dialogue to address the ethical and societal implications of neuroscientific progress.

The first chapter of Kandel’s Principles of Neural Science, the standard introductory text for neuroscience, states: “Such a unified approach, in which mind and body are not seen as separate entities, rests on the view that all behavior is the result of brain function. What we commonly call the mind is a set of operations carried out by the brain” (1) (p. 7). The view that the mind is reducible to brain function is presented as if it were a premise of neuroscience research, or at least a matter of scientific consensus. Certainly, this view is widespread: its influence can be discerned in the most controversial interpretations of neuroscience, including neuroessentialism (the idea that knowledge about the nervous system suffices to understand the human person) (2) and neurodeterminism (the idea that free will is an illusion, because neural activity fully determines our behavior) (3). It also underlies the identification of mental illness with brain disorders (4), and the understanding of consciousness as the product of brain processes (5). However, to our knowledge, no study has systematically analyzed neuroscientists’ opinions and beliefs about the mind–brain problem (i.e., the relationship between mental and neural activity) and related matters.

Before we inquire into the beliefs of neuroscientists about the mind–brain problem, perhaps we should ask whether it is relevant to their work. A brief look at the history of neuroscience indicates that mind and brain were jointly studied until recently (6), and that their relationship was matter of serious concern for preeminent figures such as William James (7), Roger Sperry (8), Charles Sherrington (9), and John Eccles (10). In recent decades, however, the mind–brain problem has been treated more often as a matter for philosophers. If the opening statement in Kandel’s textbook is taken as representative, there is no mind–brain “problem” in neuroscience because all mental activity is presupposed to be the product of brain function. However, even if this position is widely endorsed by neuroscientists, it could be interpreted to mean different things—that mental function is negligible, as proposed by reductive physicalism (11), or that mental features emerge from brain processes, as proposed by emergentism (12), which can be considered as a nonreductive form of physicalism. Especially when regarded as a methodological stance, the attempt to understand mental activity in terms of brain processes is compatible with a wide range of positions regarding the mind–brain relationship. Functionalism, for example, proposes that the mind is a set of functions that can be implemented in different substrates, biological or synthetic (13). Another alternative is dualism, which, in its strongest version (substance dualism), posits that mind and body are different entities that somehow interact in living beings (10). Finally, a more integrative perspective is dual-aspect monism, which proposes that human beings are unitary realities with two aspects, subjective and objective, so that strictly speaking we cannot separate mind from body as distinct entities (14). What do neuroscientists think about these alternatives? In the absence of any systematic study, our only resources for answering this question are opinion articles and interviews with high-profile scientists, most of whom endorse some form of reductive physicalism (see, for example refs. 15–17). However, we cannot say whether this sample is representative of the field.

Also, it should be emphasized that the opinions and beliefs of neuroscientists with respect to the mind–brain problem, whether explicit or implicit, can strongly influence a wide range of other issues. For example, a recurring topic of debate at the intersection between neuroscience and philosophy is free will (18). Do our brains determine our behavior? Is the nervous system best understood as a deterministic process? Even more fundamentally, does all science rest on a premise that nature is a “closed system” of deterministic causality? These are not just matters of philosophical debate; they have consequences for our thinking about ethical and even legal questions. Consider the question of moral responsibility: depending on their view of the mind–brain relationship, some neuroscientists argue that moral responsibility does not really exist—that guilt and culpability are just folk-psychology terms (3, 11), while others believe that this question is outside the scope of neuroscience (19).

Beliefs about the mind–body relationship also condition the views of neuroscientists about the future possibilities of neuroscience. As mentioned above, neuroessentialism holds that advances in neuroscience will eventually provide us with a complete understanding of the human being. Based on this perspective, mind reading through brain recording might one day be possible (20). Even more far-fetched is the transhumanist claim that one day we will achieve a kind of immortality through mental uploading (i.e., using synthetic devices to duplicate or “receive” human minds) (21). Less futuristic, but still in the realm of science fiction, is the suggestion that because our experience of “reality” is a brain-produced simulation, we could be “brains-in-a-vat” (22) or in a Matrix-like scenario (23). More practically, beliefs about the mind–brain relationship affect our understanding of mental illness, especially the question of whether it is entirely reducible to brain disorders. The brain-based view of mental illness might seem to be widespread among neuroscientists (4), but in fact this has never been studied.

Finally, beliefs about the mind–brain relationship condition neuroscientists’ views of what David Chalmer’s famously distinguished as the “easy” and “hard” problems of consciousness (24). The former problems refer to ongoing efforts to define the neural correlates of mental processes, including individual perceptions, thoughts, desires, and beliefs. The latter refers to the capacity of neuroscience to explain subjective experience itself, that is, the experience of redness when we look at something red (see also ref. 25).

Previous research has examined the relationship between opinions on the mind–body problem and religious belief in a Finnish sample of lay people (26), revealing an association between dualism and belief in the afterlife. Also, attendees of the 2014 Brazilian Congress of Psychiatry (about 600) were asked specific questions about the mind–brain relationship, and showed a tendency toward reductive physicalism; however, after a debate within the congress, they were asked again and showed less reductive views (27). Meanwhile, a previous study conducted in Edinburgh (of 250 university students) and Liège (1,858 people, including healthcare workers and lay people) revealed a tendency toward dualism (28). A more recent survey explored the opinions of consciousness researchers (N = 232) about specific issues of their field, excluding topics related to the mind–body relationship (29). Moving to more restricted scopes, neuroscientists (N = 312) were asked about neural “storing” of long-term memories, and the future of this specific field (30). Apart from the mind–body relationship, previous research has explored free will beliefs in relation to dualism and determinism, including a multicultural sample of lay people (N = 1,800, the United States and Singapore). A larger online video survey (N = 4,385) among “educated” people revealed an inclination toward compatibilism, that is, belief in free will alongside physical determinism (31).

The main goal of this survey is to collect neuroscientists’ opinions on topics that are critically important to their field and yet rarely discussed: the mind–brain problem, the computational paradigm of the brain, free will, neuroessentialism, and related questions about the future of neuroscience. The answers that scientists give about these topics reflect underlying systems of belief, which likely influence how their research is framed and directed (32). Also, these beliefs shape how research results are interpreted, disseminated to the public, and taught to students. In turn, these tendencies could influence research priorities and resource allocation, and even affect policy choices in areas as varied as mental health, ethics, end-of-life management, and AI.

Given these consequences, we believe that neuroscientists’ beliefs about the mind–brain relationship are best brought into the open. Although the nervous system remains the primary focus of neuroscience research, the entanglement of neural and mental activity inevitably involves neuroscientists in questions that are at once scientific and deeply philosophical. Especially in the era of the “global brain initiative,” when international projects are joining forces to increase our knowledge of the nervous system, it is essential to articulate the presuppositions of neuroscience research and allow these to be examined in the spirit of open, interdisciplinary dialogue.

More than 2,600 neuroscientists participated in the survey. Most were over forty years old, worked in Western Europe or North America, and had a background in medicine or the natural sciences. Nearly half identified as atheists or agnostics, and most considered the mind-brain problem important for neuroscience and their own research. Reductive physicalism emerged as the most widely endorsed position, although views on computationalism and determinism were more divided. Despite this, most respondents rejected the claim that the nervous system fully determines human behavior. Participants generally expressed optimism about the future of neuroscience, including its potential to explain human nature and address the hard problem of consciousness. Principal component analysis (PCA) identified five latent components, which varied according to participants’ academic background, research area, and sociodemographic, religious, and political characteristics.

## Results

**Fig. 1.**
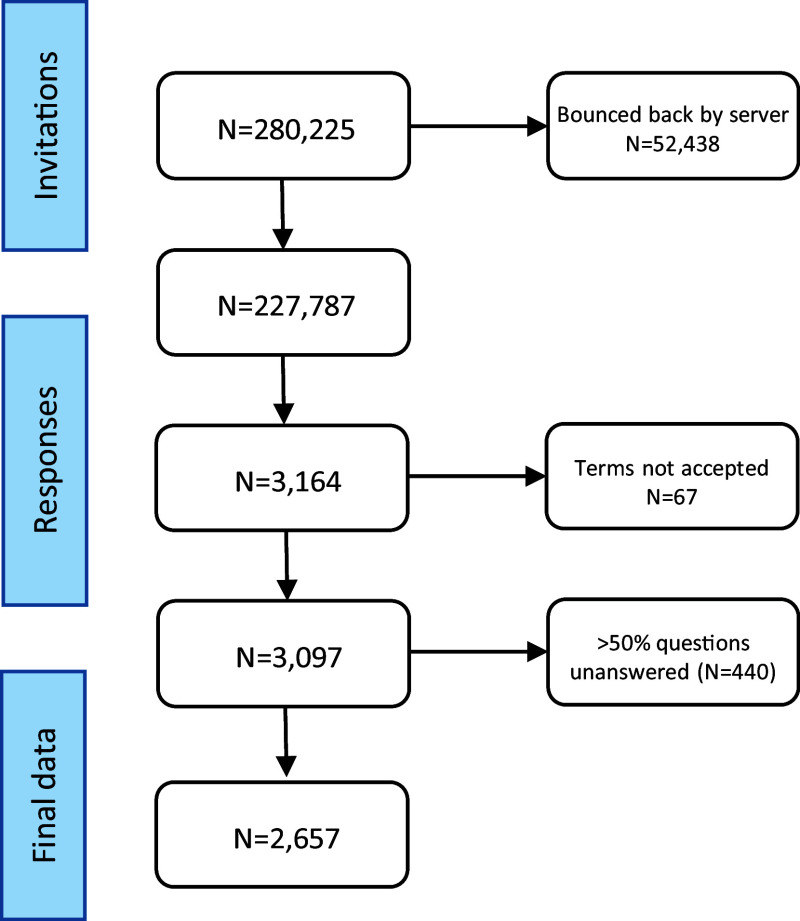
Flowchart showing the stages of data collection and the final number of responses.

The survey was sent between April and May 2025 to 280,225 researchers whose email addresses were listed in neuroscience journals (according to JCR categories) over the last 10 y. The server bounced back 52,438 invitation emails (18.7%). The invitation was responded to by 3,164 people (response rate = 1.39%), of whom 67 did not accept the terms of the survey. All questions included the option “Prefer not to answer” and were allowed to be left blank, and as a result not all questions were answered by all participants. Out of the 3,097 respondents who explicitly accepted the terms, 440 left most (>50%) of the questions unanswered or responded “Prefer not to answer”; these were excluded from the analyses. Therefore, the results shown here include a maximum of 2,657 respondents for each question (Fig. 1). The last question was answered by 2,522 participants, resulting in an attrition rate of 18.6%.

### Demographic Variables.

**Fig. 2.**
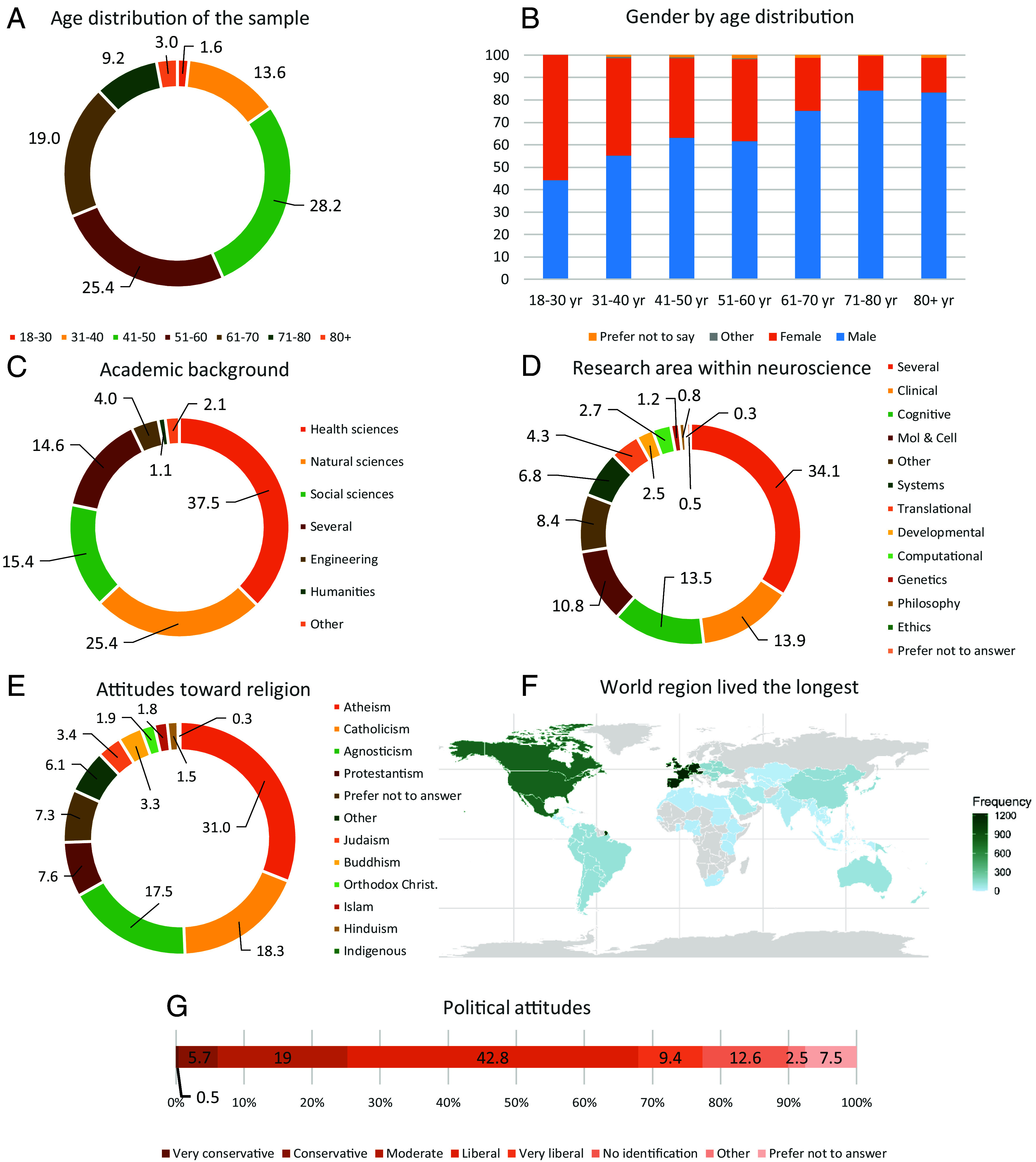
Main results of the demographic variables included in the survey. (*A*) Age distribution. (*B*) Gender by age distribution. (*C*) Academic background. (*D*) Research area within neuroscience. (*E*) Attitudes toward religion. (*F*) World region were participants declared to live the longest, color-coded by number of responses. (*G*) Political attitudes. All results except for *F* are percentages.

See Table 1 for the demographic questions included in the survey. All demographic variables are reported in the *SI Appendix*. In summary, our sample was biased toward male, middle-aged, and Western participants. Also, they were mainly trained in health and natural sciences and worked in clinical and cognitive neuroscience. Most of them were politically liberal and nonreligious (over 48% self-declared as atheists or agnostics), and the most frequent religious affiliation was Catholicism (Fig. 2). Without an official global census of neuroscientists, it remains unclear whether this is representative of the entire neuroscientific population.

**Table 1.** Demographic questions included in the survey

D1	Gender
	(Male; Female; Other; Prefer not to say)
D2	Age
	(18 to 29; 30 to 39; 40 to 49; 50 to 59; 60 to 69; 70 to 79; 80+)
D3	Which of the following best describes your academic background?
	[Health Sciences (e.g., Medicine, Nursing, Pharmacy); Natural Sciences (e.g., Chemistry, Biology, Physics); Social Sciences (e.g., Psychology, Sociology, Anthropology); Engineering (e.g., Biomedical Engineering, Computer Science); Humanities (e.g., Philosophy, Linguistics); Other (open text)]
D5	What is your primary research area in neuroscience?
	(Developmental neuroscience; Cognitive neuroscience; Molecular and cellular neuroscience; Neurogenetics; Systems neuroscience; Computational neuroscience; Translational neuroscience; Ethics and neuroscience; Neuroscience and philosophy (other than ethics); Clinical neuroscience; Other (open text); Prefer not to answer)
D8	World region where you have lived the longest
	(North America; Central America and the Caribbean; South America; Western Europe; Eastern Europe; Northern Africa; Sub-Saharan Africa; East Asia; South Asia; Southeast Asia; Central Asia; Middle East; Oceania; Other (open text); Prefer not to answer)
D9	Which of the following religions do you identify with the most?
	(Agnosticism; Atheism; Buddhism; Catholicism; Hinduism; Islam; Judaism; Orthodox Christianity; Protestant/Evangelical Christianity; Traditional/Indigenous Religion; Other (open text); Prefer not to answer)
D10	Weekly participation in religious services
	(Very involved (regular practice); Occasional; Rare; Not involved; N/A)
D11	Political orientation
	(Very conservative/voter of far-right parties; Conservative/voter of right-wing parties; Moderate/voter of parties that shift between right and left; Liberal/voter of left-wing parties; Very liberal/voter of far-left parties; I do not identify with any orientation; Other (open text); Prefer not to answer)

The options for each question are shown in parentheses, separated by semicolons. Questions D6 (world region of birth) and D7 (world region of work) have been omitted for brevity and redundancy in the analyses.

### Overall Description of the Attitudes Toward the Mind-Brain Problem, the Computational Paradigm, Free Will, and the Future of Neuroscience.

**Fig. 3.**
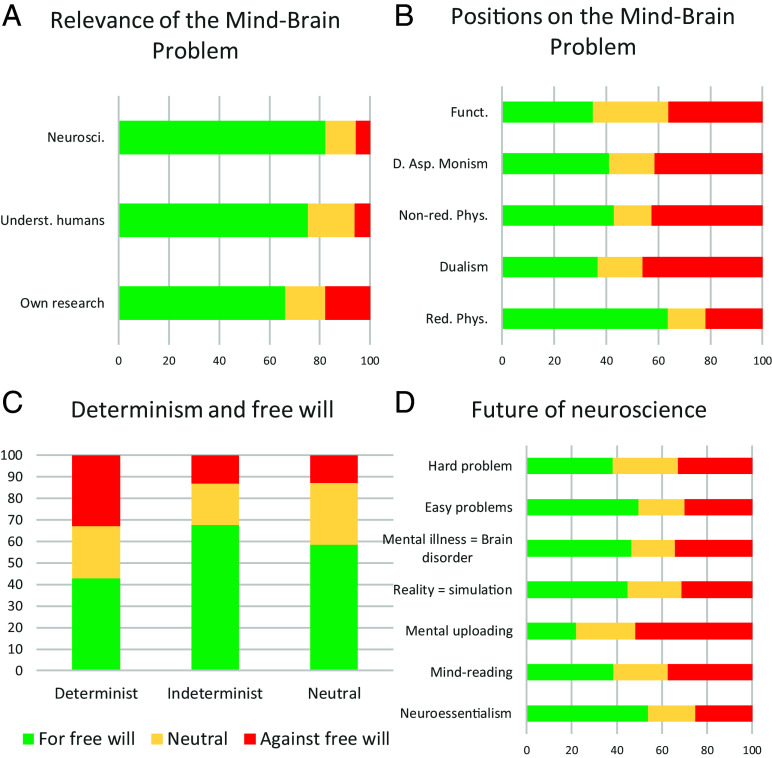
Summary of responses to most questions of the survey. “Agree” (in green) includes “partially agree” and “totally agree” responses, whereas “Disagree” (in red) includes partial and total disagreement with the proposition. “Neutral” (in yellow) corresponds to “Neither agree nor disagree” responses. (*A*) Attitudes toward the relevance of the mind–brain problem for neuroscience (*Top*), understanding humans (*Middle*) and the participant’s individual research (*Bottom*). (*B*) Level of agreement with each statement defending possible attitudes toward the mind–brain problem. See Results and Table 2 for the complete description of the propositions. (*C*) Summary of responses toward (in)determination of neural processes and free will. The bars identified as “Determinist,” “Indeterminist” and “Neutral” correspond to those participants responding 0 to 4, 6 to 10, or 5, respectively, to the question about brain processes being fully determined (0 to 4) or indetermined (6 to 10). Then, the endorsement of free will is shown for each group. For clarity, this question has been inverted: it was originally formulated as agreement with the inexistence of free will, and in this figure is shown as agreement with the existence of free will. (*D*) Attitudes toward several issues about the future of neuroscience (Table 2).

Table 2 enumerates the questions in the survey discussed in this paper (we also include all survey items with response options in a Dataset S1). Also, we provide more detailed results in the *SI Appendix*. The survey began with three questions about the importance of the mind-brain problem (defined as “the relationship between mental activity and neural activity”): how relevant it is to the participant’s actual research, its relevance to understand human beings (i.e., as an anthropological issue), and whether it is (or should be) a topic of concern for neuroscience or neuroscientists. Participants were asked to rate from 0 (totally disagree) to 4 (totally agree), with 2 indicating “neither agree nor disagree.” These results are shown in Fig. 3*A*, and in the *SI Appendix* with more detail. Most of the respondents (66.28%) agreed or strongly agreed that the mind-brain problem is relevant for their research. Agreement was stronger when they were asked whether the mind-brain problem is a relevant anthropological issue (75.2%). When asked if it is a topic of concern for neuroscience, the overall level of agreement was even higher: 82.06% agreed or strongly agreed with it.

**Table 2.** Questions about the mind-brain problem and related issues included in the survey

Relevance of the mind-brain problem	
1	The mind-brain problem—that is, the relationship between mental activity and neural activity—is relevant to my research
2	I consider the mind-brain problem to be a relevant anthropological issue
3	The mind-brain problem is (or should be) a topic of concern for neuroscience or neuroscientists
Attitudes toward the mind-brain problem	
4	All mental activity (thoughts, feelings, etc.) of the human being is reducible to the functioning of the brain
5	The human being is composed of two realities, one physical (the body) and one mental, which somehow interact with one another
6	The human being is a unitary reality, but mental activity is not entirely reducible to the functioning of the nervous system
7	The human being is a unity with two dimensions, one mental and one biological, which are distinct but inseparable
8	The human being is a set of functions that could be implemented in different physical substrates
Neurodeterminism, free will, and computational paradigm	
10	Do you believe that brain functioning (and the mental activity associated with it) is constituted by deterministic processes (fixed interactions with no possibility of unexpected variations) or indeterministic processes (unexpected or unpredictable variations)?
11	Computational theory proposes that brain function can be explained as a type of computational process. To what extent do you think computational theory is a good theoretical framework for understanding the brain?
12	The computational theory of the brain is useful for my research
13	Human beings do not have freedom in their actions, as their nervous system determines their behavior
Neuroessentialism and optimism	
14	Advances in neuroscience will enable us to fully understand the human being
15	Advances in neuroscience will allow us to read human minds by recording their brain activity
16	Advances in neuroscience will achieve “mental uploading,” meaning that a human’s mental activity can be transferred to a synthetic device
17	What we call “reality” is a simulation produced by our brain
18	Every mental illness results from a brain disorder and can be explained and treated by studying and treating the brain
19	Neuroscience is already capable, or will be in the future, of explaining the neural correlates of any mental process (desires, beliefs, decisions, etc.)
20	Neuroscience is already capable, or will be in the future, of explaining the existence of subjective experience, a topic often referred to in philosophy as the “hard problem of consciousness” (David Chalmers)
22	Considering the current state of neuroscience, what percentage of the brain (including anatomy and function) have we managed to understand?
Perceptions on the mind-brain problem in neuroscience	
23	How frequent do you think the following positions on the mind–brain problem are among neuroscientists?
23a	All mental activity (thoughts, feelings, etc.) of the human being is reducible to the functioning of the brain
23b	The human being is composed of two realities, one physical (the body) and one mental, which somehow interact with one another
23c	The human being is a unitary reality, but mental activity is not entirely reducible to the functioning of the nervous system
23d	The human being is a unity with two dimensions, one mental and one biological, which are distinct but inseparable
23e	The human being is a set of functions that could be implemented in different physical substrates

**Fig. 4.**
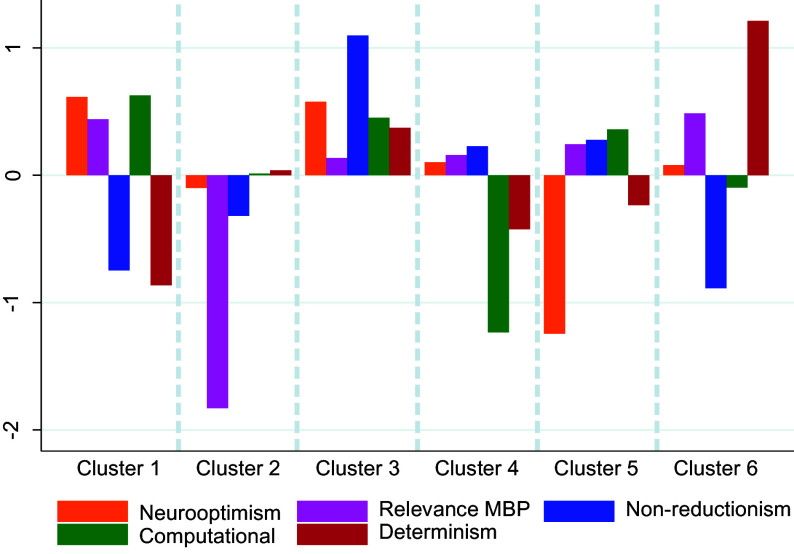
Profiles of respondents in the six-cluster solution obtained via k-means clustering. Cluster 1 represents an “eclectic” profile, with similar contributions across all components (in absolute value). Cluster 2 is characterized by a rejection of the relevance of the mind–brain problem (MBP). Cluster 3 includes nonreductionist respondents. Cluster 4 comprises participants who reject the computational paradigm of the brain. Cluster 5 reflects a rejection of optimistic expectations about the future of neuroscience. Cluster 6 encompasses respondents endorsing both determinism and reductionism.

Question numbers apply to Fig. 4. See the Dataset S1 for questions 9 and 21, which are not discussed in this report. Response to question 10 included a sliding bar between left = determinate, right = indeterminate, 5 = both. Question 11: sliding bar between 0 = poor theoretical framework, 10 = good theoretical framework. Question 22: sliding bar between 0 = we know nothing at all; 100 = we know absolutely everything. The remaining questions were answered as 0, Totally disagree; 1, Partially disagree; 2, Neither agree nor disagree; 3, Partially agree; 4, Totally agree.

Next, we showed five statements describing the most common attitudes toward the mind-brain problem: reductive physicalism, dualism, nonreductive physicalism (including emergentism), dual-aspect monism, and functionalism (Fig. 3*B*). However, these technical terms were not used (see Table 2 for the exact phrasing). As in the questions above, participants indicated their level of agreement with each statement. Overall, 63.62% agreed or strongly agreed with reductive physicalism, 36.68% with dualism, 42.94% with nonreductive physicalism, 40.94% with dual-aspect monism, and 34.69% with functionalism.

At the end of the survey, we asked participants about the frequency of these positions among neuroscientists, repeating the 5 statements expressed above about the different views on the mind-brain problem. They were asked to rate each from “very infrequent” (0) to “very frequent” (4). By doing so, we could compare their perception of neuroscientists’ opinions with the actual responses that we collected at the beginning of the survey (*SI Appendix*, Table S7). The main perception biases appeared for reductive and nonreductive physicalism: in the former, 63.62% agreed or strongly agreed with the proposition, whereas 74.39% considered it frequent or very frequent among neuroscientists; regarding the latter, 42.94% agreed or strongly agreed, but perceived agreement decreased to 33.18%.

Then, we assessed respondents’ level of agreement with the computational paradigm of neuroscience research, i.e., “that brain function can be explained as a type of computational process.” First, we asked whether this was “a good theoretical framework for understanding the brain” (on a scale of 0 to 10), and then whether the computational theory of the brain was useful for their research (on a scale of 0 to 4, as before). In the former, the average was 5.39 ± 2.57 (mean ± SD), indicating some degree of indifference toward the question. In the latter question, 1,163 participants (44.41%) agreed (responded 3 or 4) that the computational paradigm was useful for their research.

We also asked about a controversial issue involving neuroscience: whether brain functioning is constituted by deterministic or indeterministic processes (ranging from 0 = determined to 10 = indeterminate, with 5 = both) and whether human beings lack freedom because their nervous system completely determines their behavior (0 = totally disagree, 4 = totally agree). Regarding the former, 23.51% advocated deterministic processes (voted 0 to 4), and 44.37% were inclined toward indeterminism (voted 6 to 10). Regarding the statement against free will, 17.52% agreed or strongly agreed, whereas the majority of the sample (59.26%) disagreed with the proposition. See the *SI Appendix* for further analyses about this topic, including compatibilist, hard incompatibilist, hard determinist, and libertarian attitudes in the sample (33). Fig. 3*C* shows the interaction between questions about determinism and free will.

Last, we presented the participants with a set of statements that point to optimism about future advances in neuroscience, and their importance for the full understanding of human beings (see Table 2 and Fig. 3*D* for results). Here, we show the main results, which are described in detail in the *SI Appendix*.

Most respondents (53.9%) agreed or strongly agreed with the “neuroessentialist” statement that neuroscience enables/will enable a complete understanding of human beings. Support of mind reading through brain recording was moderate (38.34% agreed or strongly agreed). Meanwhile, 21.85% agreed with the possibility of mental uploading. Nevertheless, responses were more equally distributed when asking whether reality could be considered a simulation produced by our brains (44.67% agreed). Responses to the question about mental disorders being reduced to brain illness were similar (46.41%, agreed with the statement). They were slightly more optimistic about neuroscience explaining the neural correlates of any mental process (49.45% agreed). Finally, regarding the hard problem of consciousness, opinions were mixed (38.18% agreed that it would be resolved by neuroscience).

The last question asked about the percentage of the brain that neuroscientists have managed to understand so far. Participants had to answer using a sliding bar from 0 (“We know nothing at all”) to 100 (“We know absolutely everything”), with a resolution of 1. There were 2,480 responses. The mean was 31.6%, with a SD of 19.12 (median = 30, IQR = 25) (*SI Appendix*, Fig. S3).

We report the correlations between survey items in the *SI Appendix* (see also *SI Appendix*, Fig. S4).

### Latent Structure of Attitudes: A PCA.

A PCA was performed to examine the dimensional structure of the survey items (N = 2,395 excluding blank responses). We included the three questions about the relevance of the mind-brain problem, the five possible attitudes toward the mind-brain problem, the two questions about the computational paradigm, the two questions about determinism and free will, and the seven questions related to neuroessentialism and optimism. The two items with values between 0 and 10 were recoded to match the rest (between 0 and 4). Since variables were ordinal, we used polychoric correlations. The data were suitable for dimensionality reduction: the Kaiser–Meyer–Olkin measure of sampling adequacy was 0.78, and Bartlett’s test of sphericity was statistically significant [χ^2^ (171) = 15072.51, *P* < 0.001]. The parallel analysis scree plots identified five components, accounting for 59.2% of the variance. After varimax rotation, we used a loading cutoff of 0.4 (absolute value) to include items within each component (Table 3).

**Table 3.** Latent structure of the survey items after Principal Component Analysis

Item #	C1	C2	C3	C4	C5
1		0.74			
2		0.81			
3		0.87			
4	0.57		−0.51		
5			0.74		
6	−0.43		0.63		
7			0.73		
8					0.40
10					−0.58
11				0.79	
12				0.84	
13					0.73
14	0.72				
15	0.53				
16	0.45				
17					
18	0.73				
19	0.81				
20	0.76				

Main components (C1–C5) resulting from the principal component analysis, and item loadings (cutoff ± 0.40) after varimax rotation. See Table 2 for item identification.

The first component explained 18.3% of the variance and captured support for neuroessentialist/optimistic attitudes and reductive physicalism (reinforced by a negative loading of nonreductive physicalism). The second accounted for 11.1% of variance, and included the three items about the importance of the mind-brain problem. The third component captured nonreductionist attitudes (reinforced by a negative contribution of reductive physicalism), explaining 11.3% of the variance. The fourth captured support for the computational paradigm of the brain (9.4% of variance), and the fifth component encompassed determinist and functionalist attitudes (9.1% of variance).

Therefore, the PCA revealed five latent dimensions: 1) optimism/reductionism, 2) relevance of the mind-brain problem, 3) nonreductionism, 4) computational paradigm and 5) determinism. Component scores were computed for each participant and used as dependent variables in subsequent regression analyses with sociodemographic variables as explanatory variables (see next section).

To identify distinct profiles among respondents, a k-means clustering analysis was conducted using the component scores obtained from the PCA (Table 4 and Fig. 4). The optimal number of clusters was assessed using multiple validity indices. The silhouette coefficient and the Davies–Bouldin index both indicated a six-cluster solution. Cluster 1 (N = 380, 15.87% of the sample) included respondents with nonpolarized or eclectic attitudes, since all components had some representation. This group supported optimistic attitudes, was relatively interested in the mind-brain problem, tended to reject nonreductionist attitudes, supported the computational paradigm of the brain and rejected determinism, advocating for free will. Cluster 2 (N = 318, 13.28%) was characterized by respondents who rejected the relevance of the mind-brain problem. Cluster 3 (N = 446, 18.62%) included respondents with nonreductionist attitudes, whereas Cluster 4 (N = 455, 19%) grouped participants against the computational paradigm. Cluster 5 (N = 429, 17.91%) was composed of respondents who rejected neurooptimism and reductionism, and Cluster 6 (N = 367, 15.32%) was characterized by rejecting nonreductionist attitudes and supporting determinism.

**Table 4.** Sample clustering based on the latent components found after PCA

Cluster #	C1-optimism	C2-relevance MBP	C3-nonreductionism	C4-computational	C5-determinism
1	0.615	0.440	–0.746	0.626	–0.865
2	–0.100	–1.829	–0.320	0.012	0.037
3	0.576	0.135	1.097	0.450	0.371
4	0.101	0.158	0.226	–1.233	–0.424
5	–1.245	0.242	0.277	0.360	–0.234
6	0.079	0.486	–0.887	–0.098	1.211

K-clustering of the latent components found after PCA. Numbers are a proxy of the load of each component in the cluster, with 0 indicating an average load. MBP, Mind–Brain Problem.

### Sociodemographic Analysis of the Latent Components.

**Fig. 5.**
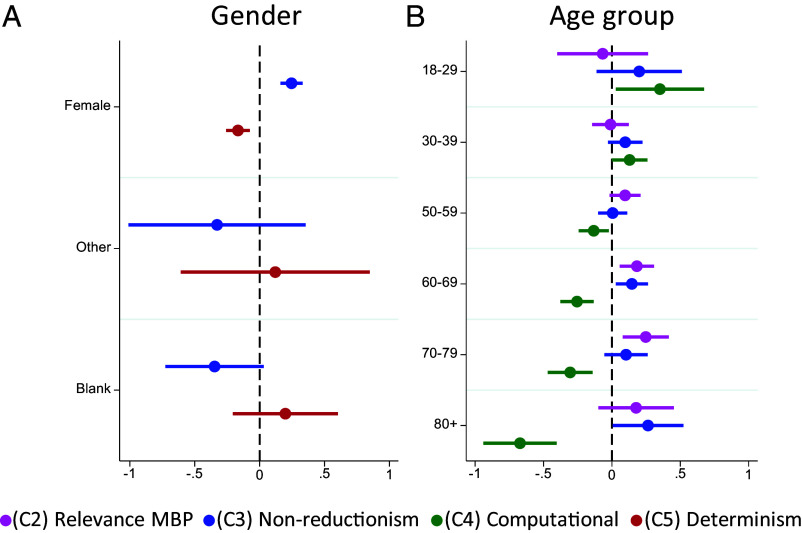
Forest plots showing significant differences in components C2–C5 across gender (*A*) and age (*B*). Component C1 is excluded because no significant differences were observed across these groups. Male and the 40 to 49 age group served as the reference. Points indicate estimates; lines represent 95% CI.

**Fig. 6.**
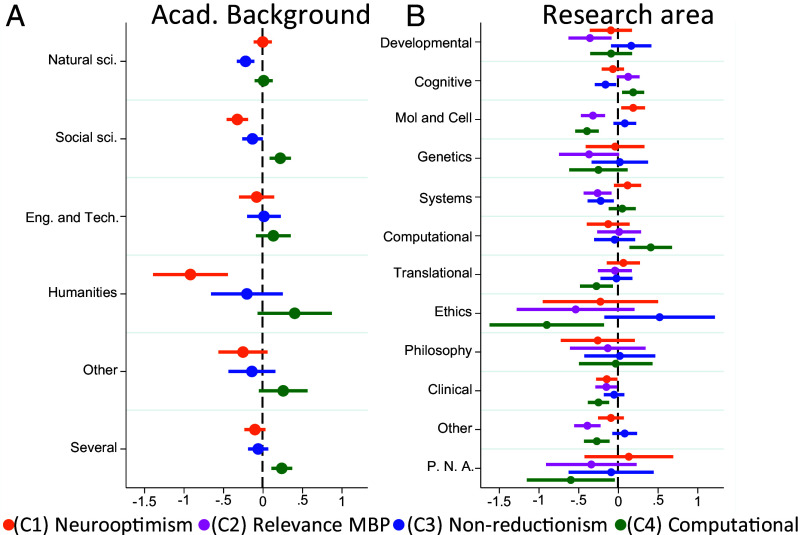
Forest plots showing the main differences in components C1–C4 across academic background (*A*) and research area (*B*). Component C5 is excluded because no significant differences were observed across these groups. Health sciences and “several” research areas were the reference groups. Points indicate estimates; lines represent 95% CI. Eng. and Tech., Engineering and Technology; Mol. and Cell., Molecular and cellular neuroscience; P.N.A., Prefer not to answer.

**Fig. 7.**
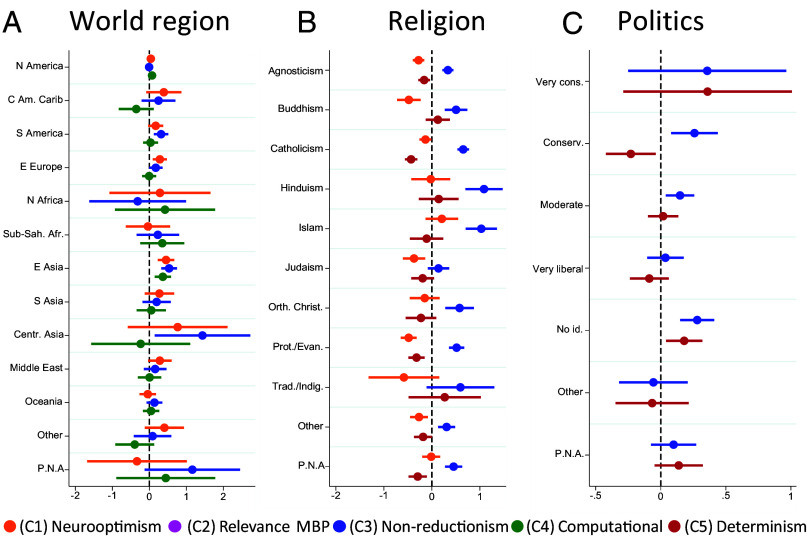
Forest plots showing the main differences in components across world region (*A*), religious attitudes (*B*), and political self-identification (*C*). Reference groups were Western Europe, atheism and liberal/left-winged voters. Points indicate estimates; lines represent 95% CI.

Finally, we ran a linear regression for each latent component, with sociodemographic variables (gender, age range, academic background, research area, world region where they lived the longest, religion, and political attitude) as predictors. The reference value for each predictor was set to the most numerous group (male, 40 to 49 y, health sciences, “several” research areas, Western Europe, atheism, and liberal, respectively). Results are described in detail in the *SI Appendix*. Figs. 5–7 summarize the results of these analyses.

The first component (showing neurooptimistic and reductionist attitudes) was more strongly endorsed by respondents working in molecular and cellular neuroscience and by those living in Eastern Europe and East Asia. However, scores were lower among participants with a background in social sciences (e.g., psychology, sociology, anthropology) and the humanities (e.g., philosophy, linguistics), working in clinical neuroscience, and with several religious attitudes: agnosticism, Buddhism, Catholicism, Judaism, Protestant/Evangelical Christianity, and “other” attitudes.

The second component (interest in the mind-brain problem) differed significantly across ages and research areas. In particular, older age groups were more interested in the topic, whereas respondents working in molecular and cellular neuroscience, systems neuroscience, and clinical neuroscience were less interested in the mind-brain problem.

The third component (nonreductionist attitudes toward the mind-brain problem) differed across nearly all sociodemographic variables. First, female participants were more nonreductionist than males. Second, older participants (particularly those in the 60 to 69 range and those older than 80) were also more nonreductionist than the reference group. Third, those with a background in natural and social sciences were more reductionist than the reference group. Fourth, we found similar attitudes (lower endorsement of nonreductionist attitudes) in cognitive and systems neuroscience. Fifth, participants from South America and East and Central Asia scored higher on this component, thus being more nonreductionist than those from Western Europe. Sixth, concerning religion, nearly all groups (all but Judaism and Traditional/Indigenous) showed stronger nonreductionist attitudes than atheism. Finally, conservatives, moderates, and those not identified with any party were significantly more nonreductionist than the reference group.

The fourth component (endorsement of the computational paradigm of the brain) showed more restricted differences. The most evident was a nearly linear decline with age range. Conversely, participants with a background in social sciences and several academic fields supported the paradigm more strongly. The same trend was found for those working in cognitive and computational neuroscience, whereas those working in molecular and cellular biology, translational, ethics, and clinical neuroscience were less supportive to the paradigm. Finally, respondents from East Asia were more favorable to the computational paradigm of the brain.

The fifth component encompassed deterministic and functionalist attitudes, and the regression analysis also revealed differences in relation to the sociodemographic variables. Remarkably, female participants scored significantly lower than males, pointing to more indeterminist and pro-free will attitudes. The same trend was found in several religious attitudes: agnosticism, Catholicism, and Protestant/Evangelical Christianity. Finally, voters of conservative parties rejected determinism more strongly than the reference group (liberal voters), and those rejecting identification with any party were more determinist.

Overall, the analyses show that philosophical attitudes among neuroscientists are shaped by sociodemographic factors, though the strength of these associations varies across components. While some dimensions—such as neurooptimism and interest in the mind–brain problem—are primarily influenced by disciplinary background, age, and geographic context, others, especially nonreductionist and deterministic views, reflect broader differences linked to gender, religion, and political orientation (Figs. 5–7). Together, these findings highlight that perspectives on the brain and mind are not only rooted in scientific training but also embedded in wider cultural and ideological frameworks.

## Discussion

Our survey provides a window onto the diverse philosophical and meta-scientific views of neuroscientists. According to our results, the mind-brain problem is widely acknowledged as a major topic of concern for neuroscience, important not only for understanding the human being as a whole, but also for respondents’ areas of research. Other significant results include general support for reductive physicalism and the computational paradigm, and an optimistic attitude about the future of neuroscience and its potential to understand the human being. Notably, we also observe an apparent tension between respondents’ acceptance of reductive physicalism and support for free will. We discuss these findings in more detail below.

Our survey of 2,657 neuroscientists reveals a significantly more reductionist and optimistic worldview than previous studies of lay and professional groups. While undergraduates often lean toward dualism or emergentism (26, 28), two thirds of our respondents endorsed reductive physicalism—a stance notably higher than the materialist views found among Brazilian psychiatrists (53%) or European healthcare workers (40%) (27, 28). Neuroscientists also display greater neurooptimism regarding consciousness; while two-thirds of specialized researchers assume a persistent “explanatory gap,” only one-third of our sample explicitly rejects the possibility of neuroscience solving the “hard problem”(29). Furthermore, our results align with recent 40% probability estimates for mind reading but remain more conservative regarding mental uploading (approximately 20% agreement compared to 62% in expert emulation estimates) (30). Our findings also support a compatibilist view of free will, mirroring trends among educated laypeople (31). Collectively, these comparisons suggest that while neuroscientists share some common intuitions, their disciplinary training fosters a distinct commitment to reductionist and optimistic explanations of the mind-brain relationship.

Participants were strongly inclined toward reductive physicalism. This was the expected result, given they are researchers dedicated to the study of brain function. However, our results show that agreement with reductive physicalism is not as widespread as neuroscientists themselves predicted: 75% of participants estimated that this position was frequent or very frequent, which is higher than the proportion of respondents who actually held this position (about 66%). This difference showed the opposite trend for nonreductive physicalism. This discrepancy is consistent with well-documented phenomena in social psychology, such as pluralistic ignorance (when group members misunderstand the attitudes or beliefs of their peers) (34) and the false consensus effect (the overestimation of the level of agreement that others have with my beliefs) (35). In our case, pluralistic ignorance only affects physicalism, since dualist, dual-aspect, and functionalist attitudes are accurately perceived. Also, false consensus effect seems to affect supporters of reductive physicalism. We can conclude from these results that neuroscientists tend to believe that mental activity is completely reducible to brain function, although actual positions are more diverse than perceived norms.

In light of this tendency, a striking result is the coexistence of strong support for reductionist attitudes with the simultaneous endorsement of free will. This pattern may be interpreted as a form of cognitive dissonance, whereby individuals hold mutually inconsistent beliefs without fully integrating them at a reflective level (36). Alternatively, respondents could implicitly distinguish between different explanatory levels, reconciling neural determinism at the biological level with notions of agency at the personal or phenomenological level. In this sense, rather than indicating simple inconsistency, these responses may reflect the coexistence of multiple conceptual frameworks, a pattern that resonates with longstanding philosophical positions such as compatibilism. Further, the high number of neutral responses to the question about the nature of brain processes suggests either acceptance of both possibilities (determination and indetermination) or uncertainty, which is consistent with ongoing debates in the field (3, 37–43). Nevertheless, the finding that neuroscientists support free will irrespective of their response to the mind-brain problem is an intriguing result, especially in light of anti-free will statements by high-profile neuroscientists (44–46).

We wish to make a few observations concerning the practical implications of our results. First, although our study suggests that an overwhelming majority—over 80%—of neuroscientists believe that the mind-brain problem is relevant to neuroscience, basic research on this topic is not promoted or supported by funding sources. This lack of support may be a consequence of the perception that the mind-brain problem is a topic for philosophical research, not a matter of serious empirical inquiry. However, many philosophers and scientists believe that the best approach to this problem will involve experts in brain anatomy and function working alongside experts from other disciplines, acknowledging both the possibilities and limitations of neuroscientific research. Proponents of “neurophenomenology” have indicated how this collaborative form of research could be carried out (47). Also, frameworks such as “spatiotemporal neuroscience” developed by Georg Northoff and others show the possibility of an integrative, nonreductive approach that combines insights from philosophy with empirical methodology (48). In principle, these interdisciplinary and integrative research do not require any substantial special equipment or funding, but they do need to be conducted in a supportive environment that facilitates dialogue and encourages creativity and risk-taking.

Second, we found a widespread “compatibilist” attitude regarding free will. As discussed extensively in academia, neural determinism would have serious legal and moral consequences if it were to be accepted by society at large (for example, 49). Based on our study, however, only a small proportion of neuroscientists accept the rejection of free will on neurobiological grounds. This result suggests to us that the center of debate should shift to more practical issues concerning the development of human agency and its impairment by specific pathologies.

Third, over half of our sample believes that neuroscience can achieve a full understanding of the human being. This attitude of neuroessentialism is in tension with the ostensible promotion of “interdisciplinarity” by brain initiatives around the globe. To solve self-contradictory attitudes, our results suggest that neuroscientists can benefit from sustained dialogue with other disciplines, including the physical sciences, on the one hand, and disciplines of humanities and social sciences, on the other. The need for such interdisciplinary dialogue is more evident than ever, as the rapid development of AI has renewed long-standing questions about the nature of intelligence and the distinction between humans and machines. Although our survey did not include questions explicitly addressing AI, understanding neuroscientists’ views on this issue would be of considerable value, and we encourage further research in this direction.

A final practical implication is the treatment of mental illness, whose prevalence seems to be increasing, with massive costs for individuals and society at large. The fact that nearly half of participants believe that mental disorders can be understood and treated solely by studying and treating the brain suggests to us a need for dialogue between neuroscience and other perspectives on mental illness, including diagnosis and treatment, as suggested by several authors (50–53).

While our study benefits from a large sample size and high statistical precision, its generalizability may be limited by several factors. The broad sampling strategy likely inflated the target population, resulting in a low response rate that may not reflect the wider neuroscience community. In addition, response bias is a key concern, as the survey framing may have preferentially attracted participants with a preexisting interest in the mind–brain problem, potentially overestimating its perceived relevance. Finally, the sample is demographically skewed toward male and senior researchers from Western Europe and North America, and to certain areas in neuroscience, which may limit generalizability. However, even if this is the case, patterns of association between questions and variables are likely more universal and still present in all samples. These limitations could motivate future research to administer the survey in groups underrepresented in our study.

In conclusion, our research shows that the mind-brain problem is indeed a topic of serious concern for neuroscientists, although response bias could possibly have amplified this result. We also found that the most accepted attitude toward the mind-brain problem is reductive physicalism, which is positively associated with neuroessentialist and neurooptimistic views. This survey is a systematic attempt to define the belief system of the international neuroscientific community. We hope that it will contribute to the development of new lines of research, both within neuroscience and in collaboration with other fields.

## Materials and Methods

### Institutional Ethical Board Approval.

This project was reviewed and approved by the University of Navarra’s Committee on Research Ethics, under protocol number 2024.293. The approval included a thorough evaluation by the IT Compliance & Data Governance of the University. All collected data were anonymous. Even though the survey was individually sent by email, we did not collect email addresses with the results.

### Survey.

The survey consisted of a set of questions thematically organized, but not intended to be a validated psychometric instrument (Tables 1 and 2 and Dataset S1 with the whole survey). It was designed by our research group, with extensive experience in the mind-brain problem and the dialogue between neuroscience and the humanities. All questions are described in detail in the Supporting Methods.

### Target Sample.

Our intention was to reach a large, diverse, and international sample to obtain representative results. Thus, we used a script that used PubMed’s Application Programming Interface (API) to collect email addresses from researchers publishing in neuroscience journals during the last ten years. After removing duplicates, we obtained a database containing 280,225 emails. Only invited researchers could answer the survey, since the access link was inactivated if it was forwarded to another person. It could be argued that not all researchers publishing in neuroscience journals are neuroscientists. However, the concept of “neuroscientist” is vague itself and a counterargument is that anybody contributing to a neuroscience article could potentially be considered one. Moreover, in the invitation email, researchers were asked to refrain from answering the survey if they did not consider themselves neuroscientists. See *SI Appendix*, *Supporting Methods* for more information.

The questionnaire was distributed using Qualtrics XM (Academic Research, user-based license) and was designed to take approximately 10 min to complete.

### Dataset Preparation and Analyses.

Statistical analyses were performed in Stata 16.1 (StataCorp LLC, College Station, TX) except for principal component and cluster analyses, which were carried out in R (R Core Team, Vienna, Austria). To examine the latent structure underlying respondents’ answers, a PCA was performed on most survey items (excluding those asking about the perceived frequency of different attitudes toward the mind-brain problem). It was used as a dimensionality-reduction technique to identify a smaller set of components capturing the main patterns of variance in the data while minimizing redundancy among the original items. Component scores for each respondent were computed from the PCA and subsequently used as input variables for the clustering analysis.

To identify groups of respondents with similar profiles across the extracted dimensions, we conducted a k-means cluster analysis using the component scores as input variables. Finally, six linear regression analyses were conducted to assess differences in each component across sociodemographic variables. Thus, the dependent variable was individual scores for each component, and regressors were gender, age range, academic background, research area, world region where they lived the longest, religion, and political attitude. The reference value for each predictor was set to the most numerous group: male, 40 to 49 y, health sciences, “several” research areas, Western Europe, atheism, and liberal, respectively. See *SI Appendix*, *Supporting Methods* for further information about these analyses.

## Supplementary Material

Appendix 01 (PDF)

Dataset S01 (XLSX)

## Data Availability

Anonymized Database and analyses scripts data have been deposited in Github (https://github.com/jbernacer/Divergent-philosophical-attitudes-of-neuroscientists) (54).
