# Supplementary material for: Divergent philosophical commitments in neuroscience: Evidence from a global survey
Source: Proc Natl Acad Sci U S A. 2026 Jul 15;123(29):e2610776123. doi: 10.1073/pnas.2610776123 (PMC13389498; doi:10.1073/pnas.2610776123)
Supplement: Supplementary file 1 — Appendix 01 (PDF) [file pnas.2610776123.sapp.pdf]

PNAS

Supporting Information for

Divergent Philosophical Commitments in Neuroscience: Evidence from a Global Survey

Fabián Navarro-Peña[1], Gonzalo Arrondo[1], Nathaniel F. Barrett[1], Francisco Güell[1], Gabriel Madirolas[2,3], José Ignacio Murillo[1], Javier Sánchez-Cañizares[1], Javier Bernacer[1,3]
[1]Mind-Brain Group, Institute for Culture and Society (ICS), University of Navarra
[2]Research Center on Animal Cognition (CRCA), Center for Integrative Biology (CBI), CNRS, Toulouse University
[3]International Center for Neuroscience and Ethics (CINET), Tatiana Foundation

Correspondence to: Javier Bernacer
Email: jbernacer@unav.es

This PDF file includes:

Supporting text
Figures S1 to S4
Tables S1 to S12
SI References

Table of contents

Supporting methods 3
Survey 3
Target sample 4
Data collection 4
Dataset preparation and analyses 5
Supporting results 6
Demographic variables 6
Attitudes towards the mind-brain problem, the computational paradigm, free will, and the future of neuroscience 6
Correlations between variables 7
Sociodemographic analysis of the latent components 8
Satisfaction with the survey 9
Discussion 10
Figures 12
Fig. S1. Histogram (normal fit in red) showing the distribution of responses about the computational theory being a good framework to understanding the brain 12
Fig. S2. Histogram (normal fit in red) showing the distribution of responses about the brain being governed by deterministic or indeterministic processes 13
Fig. S3. Histogram showing the distribution of responses about what percentage of the brain (including anatomy and function) we have managed to understand. 14
Fig. S4. Correlations between the survey questions reported in this article. 15
Tables 16
Table S1. Age distribution for the whole sample 16
Table S2. Academic background and research area of the participants 17
Table S3. World region of birth, work and where participants lived the longest 18
Table S4. Religion or religious attitude participants identified with, and weekly participation in religious services. 19
Table S5. Political self-identification of the respondents 20
Table S6. Relevance of the Mind-Brain Problem, attitudes towards it and future of neuroscience 21
Table S7. Perceived vs actual percentage of agreement with each mind-brain problem position. 22
Table S8. Effects of sociodemographic variables on component 1 (“*neurooptimism”*) 23
Table S9. Effects of sociodemographic variables on component 2 (“*relevance of the mind-brain problem”*) 25
Table S10. Effects of sociodemographic variables on component 3 (“*non-reductionism”*) 27
Table S11. Effects of sociodemographic variables on component 4 (“*computational paradigm”*) 29
Table S12. Effects of sociodemographic variables on component 5 (“*determinism”*) . 31
SI References 33

Supporting Information Text

Supporting methods

Survey

The survey consisted of a set of questions thematically organized, but not intended to be a validated psychometric instrument (see Table 1, Table 2, and supplementary datasheet with the whole survey). It was designed by our research group, with extensive experience in the mind-brain problem and the dialogue between neuroscience and the humanities. After introducing the project and informing participants about the procedure, including data protection, we asked for their consent to participate. If they refused, the survey was terminated. When they gave their consent, there was an initial block of sociodemographic questions: gender, age, academic background, years of research experience, primary research area in neuroscience, world region of birth, world region of current employment, world region to have lived the longest, religion, weekly participation in religious services, and political orientation.

After the sociodemographic inquiries, the survey started with a set of questions about the mind-brain problem. In the first one, the issue at hand is defined: "The mind-brain problem –that is, the relationship between mental activity and neural activity– is relevant to my research." Participants were instructed to indicate, from 0 to 4, their level of agreement with each statement: 0=totally disagree, 1, 2=neither agree nor disagree, 3, 4=totally agree. The second question asked about the mind-brain problem being a relevant anthropological issue, and the third, whether this topic should concern neuroscience or neuroscientists.

Next, participants were shown 5 statements that reflected different positions on the mind-brain problem, as explained in the Results sections: reductive physicalism, dualism, non-reductive physicalism (including emergentism), dual-aspect monism, and functionalism. In the survey, we did not present these tags to avoid technical terms and potential cognitive biases towards them. Note that these five positions were not mutually exclusive; participants should indicate their level of agreement with each.

The next item is not discussed in this report for the sake of brevity, and asked about the importance (from 0 = not important to 4 = critically important) of several disciplines for understanding the human being: biology, chemistry, history, neuroscience, philosophy, physics, physiology, psychology, sociology, and others. Questions 10 and 11 had a different display, showing a sliding bar from 0 to 10 to ask about the deterministic ("fixed interactions with no possibility of unexpected variations") or indeterministic ("unexpected or unpredictable variations") nature of brain functioning (from 0=determined, to 10=indeterminate, explicitly stating that 5=both), and the extent to which the computational paradigm (i.e., the theory that brain function can be explained as a type of computational process) was a good theoretical framework for understanding the brain (from 0=poor theoretical framework to 10=good theoretical framework). Questions 12 and 13 were related to the two previous ones, asking the level of agreement (again from 0 to 4) with the computational theory of the brain being useful for one's own research, and with the lack of freedom in human actions due to the determination of behavior by the nervous system (agreement with this question pointed to free will denial).

In the following questions, participants had to indicate their level of agreement (0-4) with statements about the future of neuroscience and its potential to help understand various human issues. The first reflected a neuroessentialist stance, asserting that advances in neuroscience would enable us to fully understand the human being. After that, the survey presented the possibility of reading human minds through brain recording, and achieving mental uploading (transferring human mental activity to a synthetic device). The following questions stated that reality is a simulation produced by the brain, that every mental illness results from a brain disorder, and that neuroscience is already capable or will be in the future of explaining the neural correlates of any mental process, including desires, beliefs, decisions, etc. The latter is equivalent to the 'easy problems' of consciousness, as posed by Chalmers (26). Then, the next question explicitly asked about the hard problem of consciousness, that is, the capacity of neuroscience to

explain the existence of subjective experience. The following question is not discussed in the present report, and asked whether “our knowledge of the brain can explain why we are capable of knowing and understanding reality, including the brain itself.”

The last questions were presented differently. The penultimate used a sliding bar from 0 to 100 and asked about the percentage of the brain (including anatomy and function) that neuroscience currently understands. The final question asked how frequent various positions about the mind-brain problem were among neuroscientists, and the same statements as in the questions about the attitudes towards the mind-brain problem were presented. In this case, 0=very infrequent, 1=infrequent, 2=neither frequent nor infrequent, 3=frequent, and 4=very frequent. Finally, we asked whether the survey adequately explored the fundamental themes of the mind-brain relationship (yes/no), and participants had the possibility of typing comments.

Target sample

Our intention was to reach a large, diverse, and international sample to obtain representative results. Thus, we used a script (designed and executed by the Institute of Data Science and Artificial Intelligence of the University of Navarra, DATAI, as a paid service) that used PubMed’s Application Programming Interface (API) to collect email addresses. In this context, the PubMed API lets users access PubMed data programmatically, retrieving, in this case, the email addresses included in neuroscience articles. The inclusion criteria for the journals and articles retrieved were: 1) research articles indexed in PubMed, published in neuroscience journals (defined as those journals in the Clarivate Journal Citation Report “Neuroscience” category); 2) articles published in currently active journals, including only those journals with at least 100 articles published since 2014; 3) the articles had to be published since 2014. Once the articles were selected, we extracted the email addresses included in the article metadata under the heading “Affiliations.” After removing duplicates, we obtained a database containing 280,225 emails. Since we worked with email addresses instead of authors names, researchers could receive the invitation to participate in the survey several times if they had published with different email addresses. Only invited researchers could answer the survey, since the access link was inactivated if it was forwarded to other person.

It could be argued that not all researchers publishing in neuroscience journals are neuroscientists. However, the concept of ‘neuroscientist’ is vague itself and a counterargument is that anybody contributing to a neuroscience article could potentially be considered one. Moreover, in the invitation email, researchers were asked to refrain from answering the survey if they did not consider themselves neuroscientists.

Data collection

The questionnaire was distributed using Qualtrics XM (Academic Research, user-based license) and was designed to take approximately 10 minutes to complete. To minimize order effects and response bias, selected response options were randomized. Participants could navigate backward to revise answers, proceed without responding to all items, and pause and resume the survey within a 48-hour window. The survey was administered in English and was accessible by email invitation only. The first item asked participants to indicate consent to proceed; respondents who selected “No” were exited from the survey. All responses were fully anonymized. No direct identifiers (e.g., email addresses, IP addresses) were collected, and no technical or administrative data allowed linkage of survey responses to individual participants. They were informed about this in the initial screen of the survey.

Distribution began on April 1, 2025, with an invitation sent to 1,000 contacts. Additional batches of approximately 1,000 emails were sent every 2 or 3 days until 10,000 invitations had been distributed. Then, daily batches increased to about 10,000 invitees until the cumulative total reached 100,000. Subsequent batches were scaled for 20,000 recipients per send, culminating in 280,225 overall invitations. A reminder email was sent approximately one week after each batch. The final email (a reminder to 20,000 contacts) occurred on May 27, 2025.

Dataset preparation and analyses

The raw dataset included some variables that could not be used directly for statistical analysis. As is customary in Qualtrics surveys, the initial columns reported the start and end times of survey completion, the duration, progress (only cases with a 100% value were included), and a captcha score to assess the likelihood of a bot response.

Statistical analyses were performed in Stata 16.1 (StataCorp LLC, College Station, TX, USA) except for principal component and cluster analyses, which were carried out in R (R Core Team, Vienna, Austria). Most of the results presented in this article are descriptive statistics on frequencies and average responses, with some stratification by sociodemographic variables. We also show correlational analysis using Spearman’s rho, as it is recommended for ordinal variables. Given the large number of participants, p-values are uninformative, even after Bonferroni correction for multiple comparisons. Thus, we report rho coefficients. To examine the latent structure underlying respondents’ answers, a principal component analysis (PCA) was performed on most survey items (excluding those asking about the perceived frequency of different attitudes towards the mind-brain problem). Given the ordinal nature of the variables, the analysis was based on a polychoric correlation matrix. The two questions that had values between 0 and 10 (about determinism and indeterminism in brain processes and about the computational paradigm) were recoded to 0-4: values 0 and 1 were coded as 0; 2, 3 and 4 were set to 1; 5 was recoded as 2; 6, 7 and 8 were coded as 3; and 9 and 10 were set as 4. PCA was used as a dimensionality-reduction technique to identify a smaller set of components capturing the main patterns of variance in the data while minimizing redundancy among the original items. To determine the number of components to retain, a parallel analysis was conducted using the *fa.parallel()* function from the R *psych* package. The procedure compares the eigenvalues obtained from the observed polychoric correlation matrix with those generated from randomly simulated datasets of the same dimensionality and sample size. Inspection of the parallel scree plot indicated that the observed eigenvalues exceeded the simulated eigenvalues up to the fifth component; therefore, a five-component solution was retained. This explained 59.2% of data variance. To facilitate interpretability, a varimax rotation was applied, yielding orthogonal components and a simplified structure in which each variable loads more clearly onto a single component. As indicated in the Results section, we selected a cutoff of $\pm$0.40 to retain items within components. Component scores for each respondent were computed from the PCA and subsequently used as input variables for the clustering analysis.

To identify groups of respondents with similar profiles across the extracted dimensions, we conducted a k-means cluster analysis using the component scores as input variables. This approach groups observations based on their proximity in the reduced multivariate space derived from the PCA. The optimal number of clusters was assessed using multiple validity indices: elbow method, silhouette, Calinksi-Harabasz, and Davies-Bouldin. The elbow method was inconclusive, since no slope change was clearly visible. Both the silhouette method and Davis-Bouldin analysis pointed to an optimal 6-cluster solution, whereas the Calinsky-Harabasz test pointed to two clusters. Given the goodness-of-fit and conceptual clarity of the 6-cluster solution, we selected it.

Finally, six linear regression analyses were conducted to assess differences in each component across sociodemographic variables. Thus, the dependent variable was individual scores for each component, and regressors were gender, age range, academic background, research area, world region where they lived the longest, religion, and political attitude. The reference value for each predictor was set to the most numerous group: male, 40-49 years, health sciences, ‘several’ research areas, Western Europe, atheism, and liberal, respectively.

Supporting results

Demographic variables

See Table 1 and datasheet with the whole survey for the demographic questions included in the survey. Concerning gender, 1,754 participants identified themselves as male (66.19%), 855 as female (32.26%), 9 as “other” (0.34%), and 32 responded “Prefer not to say” or left the question blank (1.21%). The age range was biased towards middle-aged and elderly respondents (Figure 2A), likely because it was sent solely to authors who provided email addresses (i.e., typically senior and corresponding authors). See Table S1 for age distribution in detail.

We also asked about academic background and research area within neuroscience. Frequencies are shown in Table S2.

Concerning the world region of birth, work, and where they lived the longest, the majority of respondents were born, worked and lived the longest in Western Europe and North America. Table S3 shows the number of participants and the frequencies of these three variables.

We also asked about what religion (or religious attitude) the respondents identified with. Nearly half of them identified as atheists or agnostics. Next, they were asked about their weekly participation in religious services. Table S4 shows the frequencies and percentage of each religion or attitude towards religion, and also the weekly participation percentage for each religion.

Finally, we asked about their political attitudes, spanning from “very conservative” to “very liberal.” More than half of the sample identified themselves as liberal or very liberal, as shown in Table S5.

Attitudes towards the mind-brain problem, the computational paradigm, free will, and the future of neuroscience

After the demographics, we asked about the importance of the mind-brain problem to the respondent’s actual research, to understand human beings, and whether it is (or should be) a topic of concern for neuroscience or neuroscientists. Table S6 shows the responses for these questions and some of the following.

Next, we asked participants about their level of agreement with several sentences describing the most common attitudes towards the mind-brain problem, as follows: “All mental activity (thoughts, feelings, etc.) of the human being is reducible to the functioning of the brain” (reductive physicalism); “The human being is composed of two realities, one physical (the body) and one mental, which somehow interact with one another” (dualism); “The human being is a unitary reality, but mental activity is not entirely reducible to the functioning of the nervous system” (non-reductive physicalism); “The human being is a unity with two dimensions, one mental and one biological, which are distinct but inseparable” (dual-aspect monism); “The human being is a set of functions that could be implemented in different physical substrates” (functionalism) (see Table S6).

At the end of the survey, we asked participants how frequently these positions were held among neuroscientists, and we repeated the 5 statements above about the different views on the mind-brain problem. They were asked to rate each from “very infrequent” (0) to “very frequent” (4). By doing so, we could compare their perception of neuroscientists’ opinions with the actual responses that we collected at the beginning of the survey. The two main differences appeared for reductive and non-reductive physicalism. Detailed results are shown in Table S7.

After asking about positions on the mind-brain problem, we inquired about the computational paradigm (“that brain function can be explained as a type of computational process”). First, participants had to indicate how adequate this paradigm was for understanding the brain (from 0 to 10), and then whether it was useful for their research (0=totally disagree… 4=totally agree). Regarding the latter question, 306 participants (11.68%) responded 0, 426 (16.27%) responded 1, 724 (27.64%) were neutral, 759 (28.98%) agreed, and 404 (15.43%) strongly agreed that the computational paradigm was useful for their research. Figure S1 shows the histogram for the first question.

The next two questions were about determinism and free will. First, we asked participants’ opinions about whether brain functioning is constituted by deterministic or indeterministic processes (from 0=determined to 10=indeterminate, 5=both); then, whether

human beings lack freedom in their actions because the nervous system determines behavior. Figure S2 shows the histogram for the former question.

Concerning the question about humans lacking free will as behavior is determined by the nervous system, 668 participants (26.32%) strongly disagreed with the proposition, 861 (32.94%) disagreed, 607 (23.22%) neither agreed nor disagreed, 319 (12.2%) agreed, and 139 (5.32) strongly agreed with it.

The combination of both issues (determinism and the existence of free will) configures the attitudes towards neuroscience and free will: compatibilism (assuming determinism but believing in free will), hard incompatibilism (denying free will because nature is fully indeterministic, and therefore humans cannot self-determine and be responsible of their actions), hard determinism (denying free will because of determinism), and libertarianism (accepting free will by denying determinism) (1). Considering our results, 254 respondents (10% of all respondents to both questions, 2,502) appeared to be compatibilist: they disagreed or strongly disagreed with denying free will but advocated deterministic processes in brain functioning (responded 0-4 in the question about determinism). On the other hand, 136 (5.4%) participants endorsed neural indeterminacy (responded 6-10) and denied free will, supporting the view of hard incompatibilism. Only 194 participants (7.7%) were hard-determinists (agreed or strongly agreed with the anti-free will question, and voted 0-4 to question 10), and the highest percentage (776 responders, 31%) endorsed indeterminacy and free will (voted 6-10 and disagreed or strongly disagreed with question 13), so they were libertarians. Regarding respondents being ambiguous with determinism (i.e., voting 5 to question 10: 799, 32%), 58.4% disagreed with denying free will, 28.8% neither agreed nor disagreed, and less than 13% disbelieved in free will. Finally, of those respondents who were ambiguous with the existence of free will (voting 2 to question 13: 573, 23%), 40% voted 5 to question 10. Cumulatively, 25% voted less than 5, assuming determinism, and 35% assumed indeterminism. See Figure 3C for an illustrative summary of the questions about determinism and free will.

Lastly, we asked participants a set of questions about the future possibilities of neuroscience, which can be understood as 'neurooptimistic' and 'neuroessentialist' attitudes. These questions were presented as follows: a) "Advances in neuroscience will enable us to fully understand the human being"; b) "Advances in neuroscience will allow us to read human minds by recording their brain activity"; c) "Advances in neuroscience will achieve 'mental uploading,' meaning that a human's mental activity can be transferred to a synthetic device"; d) "What we call 'reality' is a simulation produced by our brain"; e) "Every mental illness results from a brain disorder and can be explained and treated by studying and treating the brain"; f) "Neuroscience is already capable, or will be in the future, of explaining the neural correlates of any mental process (desires, beliefs, decisions, etc.)"; g) "Neuroscience is already capable, or will be in the future, of explaining the existence of subjective experience, a topic often referred to in philosophy as the 'hard problem of consciousness' (David Chalmers)". See Table S6 for detailed results.

Figure S3 shows the histogram of responses to the question about the percentage of the brain that we currently understand.

Correlations between variables

We explored the associations among the questions mentioned above, excluding the demographic questions and the questions about the perception of the mind-brain problem attitudes among neuroscientists. Thus, we built a 20-by-20 correlation matrix. Since most of them were ordinal, ranging from 0 to 4, we used Spearman's rank correlation to assess the strength of the associations. We consider an absolute value of 0.5 as a proxy of a strong association, and between 0.3 and 0.5 as a moderate association. As expected, the three questions about the relevance of the mind-brain problem were correlated among themselves (see Figure S4), and only the statement about the relevance for one's own research was associated with another question apart from these, namely, the usefulness of the computational paradigm (rho = 0.296). Regarding the positions on the mind-brain problem, the strongest associations were between the physicalist reductionist and non-reductionist interpretations (-0.649), and between the dualist and the dual-aspect monist option (0.501). The reductionist stance was positively correlated with some of the optimistic and neuroessentialist statements, especially with those about mental illness (0.378), the explanation of the brain correlates of any mental process (0.363), and the

solution to the hard problem of consciousness (0.354). Although associations were weaker, the dual-aspect monist stance was negatively correlated with most of these statements. Functionalism, as expected, showed a moderate correlation with mental uploading (0.323).

There was also a strong association between the two questions assessing the reliability and usefulness of the computational paradigm (0.549). Even though rho values are slightly below 0.3, the reliability of the computational paradigm was correlated with most of the optimistic and neuroessentialist propositions. This was also the case for the usefulness of the paradigm (as individually perceived) and mind-reading (0.249). The questions about deterministic processes and free will were correlated, albeit less strongly than expected (-0.245). Concerning the final questions, the neuroessentialist statement about neuroscience being able to fully understand the human being was strongly associated with mind reading (0.369), mental uploading (0.295), mental illness as brain disorders (0.374), and the possibility of elucidating the neural correlates of any mental process (0.457) and the hard problem of consciousness (0.431). The mind-reading question strongly correlated with mental uploading (0.533), and with the elucidation of the brain correlates of any mental activity (0.331) and the hard problem (0.325). These were also correlated with mental uploading (0.331 and 0.325, respectively), and the statement about mental illness (0.499 and 0.395). Finally, as expected, the two items about the easy and hard problems of consciousness were strongly correlated (0.661).

Sociodemographic analysis of the latent components

To assess the influence of the sociodemographic variables on each latent component (see Results for a description of the principal component analysis and its outcome), we ran one linear regression for each component, including as predictors gender, age range, academic background, research area, world region where they lived the longest, religion, and political attitude. The reference value for each predictor was set to the most numerous group (male, 40-49 years, health sciences, 'several' research areas, Western Europe, atheism, and liberal, respectively).

The first regression (F(58,2125)=3.91, p<0.0001, adjusted R^2=0.0720) revealed differences in optimistic/reductionist attitudes in academic background, research area, world region, religion, and age range. Participants with a background in the social sciences (t=-4.70, p<0.001) and the humanities (t=-3.81, p<0.001) showed less optimistic attitudes than those in the reference group (health sciences). Regarding research area, there were subtle effects for those working in molecular and cellular neuroscience (t=2.44, p=0.015) and clinical neuroscience (t=-2.14, p=0.033), being the former more optimistic and the latter less optimistic than those working in several areas. About the geographical world region where participants had lived the longest, participants from Eastern Europe (t=2.90, p=0.004) and East Asia (t=3.97, p<0.001) were more neurooptimistic than those from Western Europe. Finally, several religious attitudes were significantly less optimistic/reductionist than atheism: agnosticism (t=-4.55, p<0.001), Buddhism (t=-3.84, p<0.001), Catholicism (t=-2.08, p=0.038), Judaism (t=-3.14, p=0.002), Protestant/Evangelical (t=-5.72, p<0.001), and 'other' (t=-2.83, p=0.005). See Table S8 for detailed results.

The second regression (F(58,2125)=2.63, p<0.0001, adjusted R^2=0.0415) showed differences in the relevance of the mind-brain problem across age ranges and research areas, with subtle differences in academic background and religion. More specifically, participants working in developmental (t=-2.59, p=0.010), molecular and cellular (t=-4.13, p<0.001), systems (t=-2.93, p=0.003) and clinical (t=-2.18, p=0.030) neuroscience were less interested in the mind-brain problem than the reference group (several research areas). Regarding age, older age groups (60-69 years: t=2.85, p=0.004; 70-79: t=2.88, p=0.004) were more interested in the topic. About academic background and religion, the only differences were 'other' academic background (t=2.13, p=0.034), and Islam (t=-2.35, p=0.019). Note that the former showed more interest than the baseline group (health sciences), and the latter less interest than in atheism. Table S9 shows results in detail.

The third regression assessed the non-reductionist component, yielded more significant results, and explained more variance (F(58,2125)=8.59, p<0.0001, adjusted R^2=0.1678). There were differences in nearly all sociodemographic variables: gender, academic background, research area, world region, religion, political attitudes, and, marginally, age group. Regarding

gender, female participants were more non-reductionist than male ($t=5.64$, $p<0.001$). Conversely, respondents with a background in natural sciences ($t=-3.93$, $p<0.001$) and social sciences ($t=-2.02$, $p=0.044$) were more reductionist than the reference group (health sciences). The same result was found when comparing researchers on cognitive neuroscience ($t=-2.38$, $p=0.017$) and system neuroscience ($t=-2.66$, $p=0.008$) with the baseline (several areas). Participants from South America ($t=3.13$, $p=0.001$), East ($t=4.92$, $p<0.001$) and Central ($t=2.18$, $p=0.030$) Asia had a stronger non-reductionist attitude than those from Western Europe. Concerning religion, nearly all groups showed stronger non-reductionist attitudes than participants assuming atheism: agnosticism ($t=5.73$, $p<0.001$), Buddhism ($t=4.21$, $p<0.001$), Catholicism ($t=10.59$, $p<0.001$), Hinduism ($t=5.49$, $p<0.001$), Islam ($t=6.21$, $p<0.001$), Orthodox Christianity ($t=3.80$, $p<0.001$), Protestant/Evangelical ($t=6.37$, $p<0.001$), Other ($t=3.40$, $p<0.001$), and Prefer not to answer ($t=4.08$, $p<0.001$). Regarding political attitudes, conservative ($t=2.66$, $p=0.008$), moderate ($t=2.66$), and those not identified with any orientation ($t=4.19$, $p<0.001$) were also more convinced with non-reductionism than voters to liberal parties. About age range, only participants in the 60-69 range ($t=2.43$, $p=0.015$) and older than 80 ($t=2.00$, $p=0.046$) were significantly more non-reductionist than the reference group (40-49 years old). Table S10 shows detailed results.

The fourth regression analysis ($F(58,2125)=5.43$, $p<0.0001$, adjusted $R^2=0.1053$) showed differences in the support to the computational paradigm in age range, academic background, research area, and more restrictedly in world region and political attitudes. Concerning age, support to the computation paradigm clearly declined with age: 18-29, $t=2.13$, $p=0.033$); 30-39: $t=1.95$, $p=0.052$; 50-59: $t=-2.37$, $p=0.018$; 60-69: $t=-4.10$, $p<0.001$; 70-79: $t=-3.65$, $p<0.001$; 80+: $t=-4.91$, $p<0.001$. Participants with a background in social sciences ($t=3.17$, $p=0.002$) and several areas ($t=3.51$, $p<0.001$) supported the paradigm more strongly than the baseline (health sciences). The same trend was found for those working on cognitive ($t=2.62$, $p=0.009$) and computational ($t=2.98$, $p=0.003$) neuroscience, whereas neuroscientists working on molecular and cell biology ($t=-5.24$, $p<0.001$), translational ($t=2.61$, $p=0.009$), ethics ($t=-2.45$, $p=0.014$), clinical neuroscience ($t=-3.68$, $p<0.001$), and other fields within the area ($t=-3.31$, $p=0.001$) were less supportive to the paradigm compared with the reference group (several research areas). Respondents from East Asia ($t=3.24$, $p=0.001$) showed a stronger acceptance of the computational paradigm with respect to the reference group (Western Europe), and voters to very conservative parties (t=-2-20, $p=0.028$), and refusing identification with any orientation ($t=-2.54$, $p=0.011$) showed a lower acceptance of the paradigm. Table S11 shows detailed results.

Finally, the fifth regression revealed differences in the determinist latent component ($F(58,2125)=2.94$, $p<0.0001$, adjusted $R^2=0.0490$). These differences appeared in gender, religion, politics, and marginally in age range, academic background, and world region. Female participants were clearly less deterministic than males ($t=-3.58$, $p<0.001$). Regarding religion, agnostics ($t=-2.57$, $p=0.010$) and those identifying with Catholicism ($t=-6.58$, $p<0.001$), Protestant/Evangelical Christianism ($t=-3.70$, $p<0.001$) were also stronger supporters of indeterminism and free will compared with atheism. About politics, conservative ($t=-2.36$, $p=0.018$) voters were also less deterministic; however, those rejecting identification with any political orientation ($t=2.52$, $p=0.012$) showed stronger deterministic attitudes than the reference group (liberal voters). Marginal differences appeared in the 30-39-year-old group ($t=1.97$, $p=0.049$), participants with a background in engineering and technology ($t=-2.05$, $p=0.041$), and respondents from Eastern Europe ($t=1.98$, $p=0.048$) and Northern Africa ($t=2.16$, $p=0.031$). Table S12 shows the whole results.

Satisfaction with the survey

The last question was whether the survey "adequately explored the fundamental themes of the mind-brain relationship," and allowed to post comments. Two-thirds of participants (1,679, 66.57%) responded affirmatively, and 843 (33.43%) responded negatively (135 left the question blank). Three hundred and sixteen of the former and 474 of the latter left a comment (and 14 of those who left the question blank). These numbers exclude uninformative text such as "no comment" or acknowledgments of inclusion in the survey. A detailed analysis of the comments is beyond the reach of this article. Overall, the philosophical dimension of the survey was praised by some respondents and despised by others. Positive respondents valued its intellectual challenge; negative respondents questioned its conceptual coherence. Technical and clarity issues

appeared in both groups, but were secondary to conceptual reactions. This indicates that the survey successfully stimulated reflection on the mind–brain problem, while also revealing the need for more transparent communication across scientific and philosophical audiences.

Discussion

Besides the topics discussed in the main text, we would like to add some additional remarks that could be useful to interpret our study.

About the representativeness of our results, this survey was sent to all scientists who included their email address in 'neuroscience' publications –as categorized by PubMed– in the last decade, accounting for over 280,000 invitations sent. Therefore, the target population was likely overinflated, and the response rate (about 1.4%) may not reflect the representativeness of the results. With a sample size of nearly 3,000 respondents, the study provides a high level of statistical precision, with an approximate margin of error below ±2% for proportion estimates. In any case, the most important limitation for the generalizability of our results is response bias. Because the survey was presented as a "Research project about neuroscience and the mind-brain problem," neuroscientists who recognize the importance of the mind-brain problem in neuroscience may have been more likely to respond. Thus, agreement among neuroscientists about the relevance of the mind-brain problem—for neuroscience, for understanding the human being, and for one's own research—could be lower than our results suggest. Apart from that, collected results were gender-biased towards male participants and, as expected given the methodology for selecting candidate respondents, towards more experienced scientists, as corresponding authors are usually senior researchers.

About the clarity of the survey, given the philosophical nature of many of the items included, it could be that the phrasing was not clear for some participants. In that case, a high percentage of 'neither agree nor disagree' responses would be expected. This was the result for the functionalist question (29% of responses were neutral). However, this type of response accounted for a lower percentage (between 14 and 18%) in other questions about the mind-brain problem, suggesting greater confidence in statement interpretation.

In relation to the question about the determined or indetermined nature of brain processes, it is noteworthy the high proportion of respondents that chose 5 (between 0 and 10). This would point to the acceptance of both types of processes, but also to the lack of knowledge or interest in the issue. Note that this question involves physics, which was considered as the least important discipline for understanding human beings (see question 9 in supplementary datasheet with the whole survey, whose results are not discussed in this manuscript), even compared to the social sciences and humanities (history or sociology, for example). This suggests that neuroscientists overlook the importance of physics for debates about determinism and, by extension, the free will.

Responses to questions about the current and future state of neuroscience should be interpreted through the lens of our finding that neuroscientists generally believe that we currently understand only 30% of the brain. Clearly, this is not a matter of scientific consensus, as this percentage cannot be empirically justified (because we cannot estimate what is unknown). This figure should be interpreted not as a precise empirical estimate but as an expression of widespread uncertainty about the current state of knowledge. With this result in mind, responses to the final set of questions must be interpreted as expressions of optimism about the future of neuroscience rather than predictions based on the current state of the field. Among these responses, it is noteworthy that most respondents embraced neuroessentialism (2) by supporting the view that advances in neuroscience will eventually enable us to fully understand the human being. This response implies that neuroscientists are confident that their field can advance without engaging in dialogue with other disciplines. Whereas mind reading via brain recordings appears to be possible with brain-machine interfaces (3), to our knowledge there is no scientific evidence that supports the possibility of mental uploading. It would seem, then, that the current state of the field supports those who argue against its plausibility (4). In that light, it is surprising that 22% of participants believe that mental uploading is possible, and that even 26.4% remained neutral about the statement. Also noteworthy is our finding of massive agreement with the

statement that ‘reality’ could be a simulation produced by the brain. This endorsement of brain-based constructivism tends to go hand in hand with neuroessentialism and could be interpreted in different ways. A stronger version, which we could call ‘radical’ constructivism, allows that we could be brains in vats or trapped in a Matrix-like scenario, without access to reality (5). The softer version of constructivism holds that our experience is constructed by the brain in a way that limits access but still allows for us to have knowledge of reality (6). Our intention was to detect the stronger of these positions, but we recognize that respondents who support constructivism could have the softer version in mind. Furthermore, over 46% of the sample considered mental illness to be reducible to brain disorders. A number of prominent researchers have advocated for a more holistic view of mental conditions (7–10), in which knowledge of brain impairments is integrated with a phenomenological understanding of the illness and its personal and social context. Given the increasing prevalence of these disorders despite decades of neuroscientific research, we believe that the promotion of a more interdisciplinary approach should be encouraged.

Our results indicate at least two areas in which increased support for interdisciplinary dialogue would be especially helpful. One is the surprising number of neuroscientists who endorse the possibility of mental uploading despite a complete lack of evidence and strong arguments against its plausibility. Such ungrounded claims need to be critiqued from a scientific standpoint, of course, but they also indicate a need for a more broad-based analysis and critique of the social, cultural, and economic forces that shape contemporary neuroscience and its public reception.

Finally, regarding the satisfaction with the survey, about 2,480 participants responded to the final question, which is nearly all (over 93%) of the respondents that were included in our evaluation of results (i.e., those who met the criterion of having answered at least 50% of questions). Responses to this question were not associated with attitudes towards the mind-brain problem, the computational paradigm, determinism, or free will (all rho values below 0.1). Correlations with the remaining questions (14-21) were below 0.2. Therefore, the last question does not depend on other views or attitudes assessed by the survey.

Figures

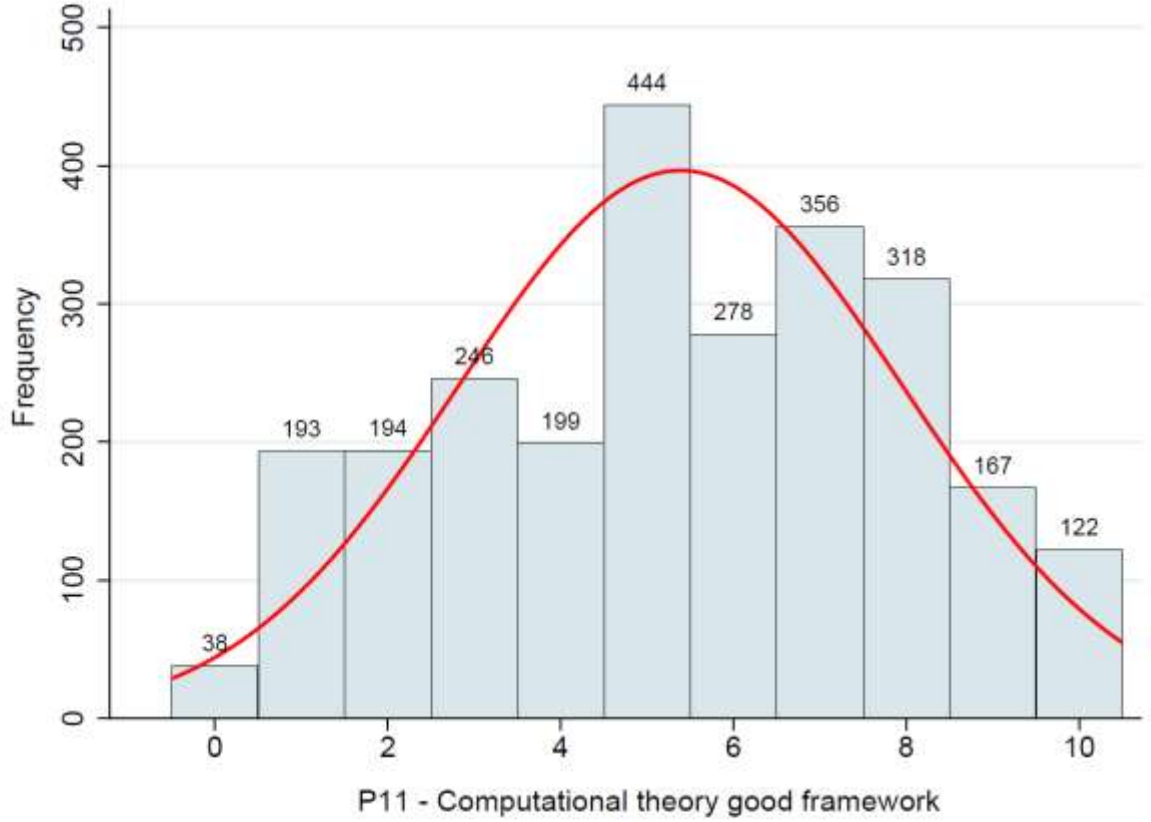

Fig. S1. Histogram (normal fit in red) showing the distribution of responses about the computational theory being a good framework to understanding the brain

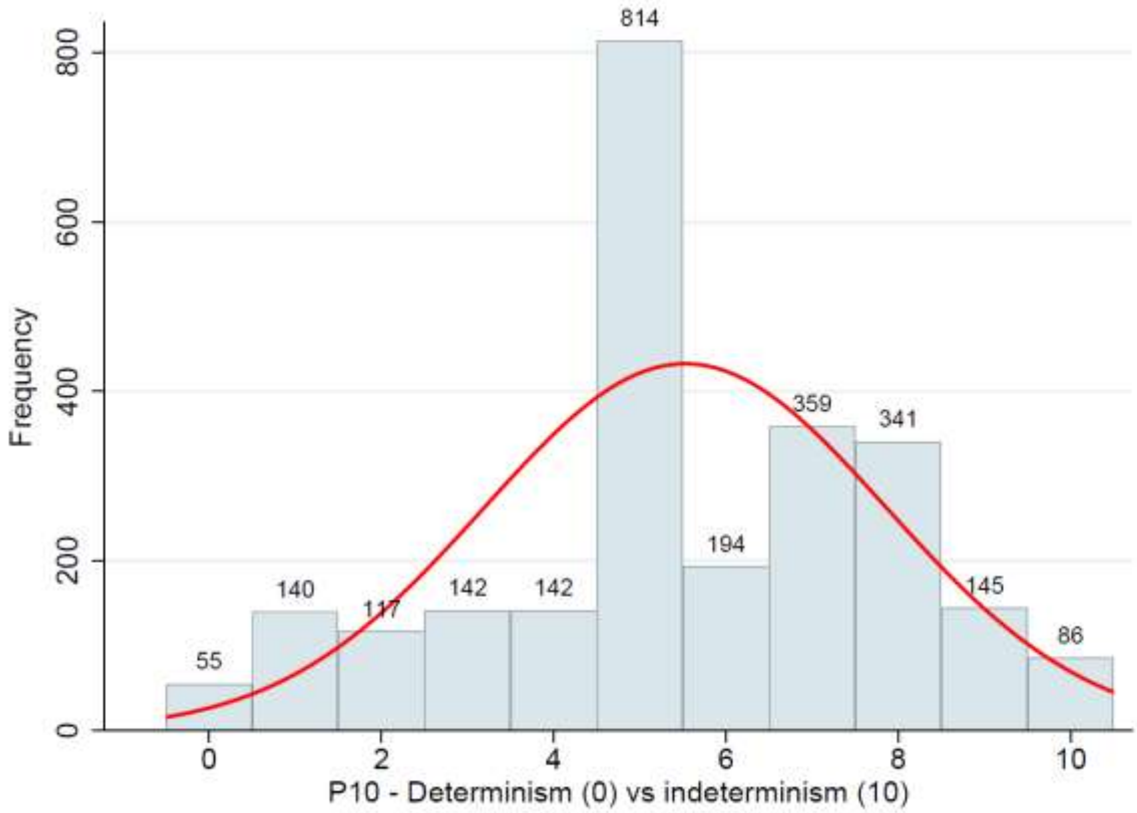

Fig. S2. Histogram (normal fit in red) showing the distribution of responses about the brain being governed by deterministic or indeterministic processes

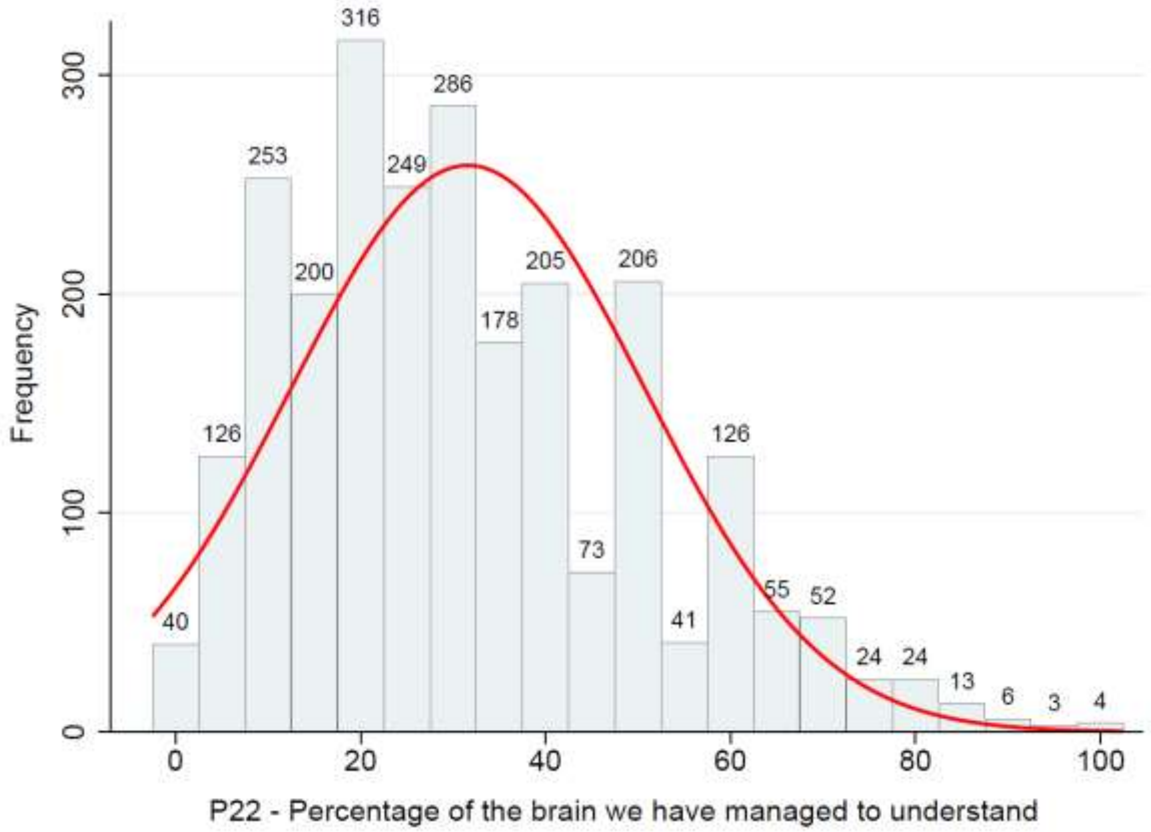

Fig. S3. Histogram showing the distribution of responses about what percentage of the brain (including anatomy and function) we have managed to understand.

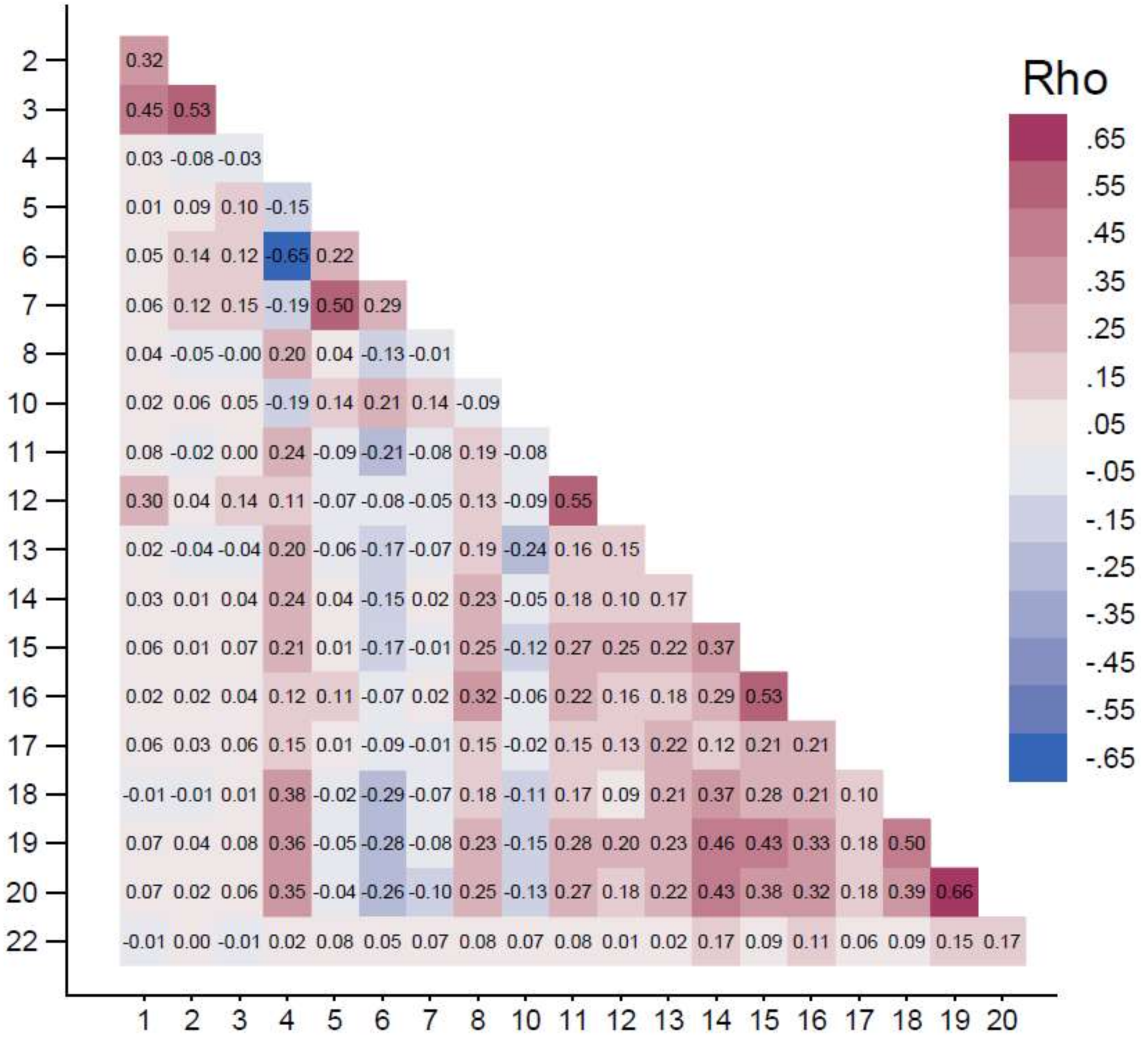

Fig. S4. Correlations between the survey questions reported in this article.
See Results and Table 2 for identification of each question. Data included in the grid are Spearman’s rho coefficients.

Tables

Table S1. Age distribution for the whole sample

Age range	N	Percent
18-29	43	1.63
30-39	357	13.57
40-49	743	28.24
50-59	668	25.39
60-69	501	19.04
70-79	241	9.16
80+	78	2.96

Table S2. Academic background and research area of the participants

Academic background	N	Percent
Health sciences	994	37.5
Natural sciences	673	25.39
Social sciences	409	15.43
Engineering and Technology	106	4
Humanities	28	1.06
Other	55	2.07
Several	386	14.56
**Research area**	**N**	**Percent**
Developmental	65	2.45
Cognitive	359	13.53
Molecular and Cell Biology	287	10.81
Genetics	32	1.21
Systems neuroscience	180	6.78
Computational	72	2.71
Translational	115	4.33
Ethics	8	0.3
Philosophy	22	0.83
Clinical	370	13.94
Other	224	8.44
Prefer not to answer	14	0.53
Several	906	34.14

Table S3. World region of birth, work and where participants lived the longest

World region	N (birth)	% (birth)	N (work)	% (work)	N (lived)	% (lived)
N. America	633	23.97	845	33.63	745	30.45
Central America/ Caribbean	25	0.95	10	0.40	16	0.65
S. America	142	5.38	77	3.06	105	4.29
W Europe	1225	46.38	1169	46.52	1140	46.59
E Europe	211	7.99	99	3.94	130	5.31
N Africa	17	0.64	3	0.12	5	0.20
Sub-Saharan A.	21	0.80	10	0.40	13	0.53
E Asia	140	5.30	98	3.90	102	4.17
S Asia	55	2.08	24	0.96	32	1.31
Central Asia	4	0.15	6	0.24	3	0.12
Middle East	58	2.20	36	1.43	47	1.92
Oceania	83	3.14	117	4.66	91	3.72
Other	17	0.64	13	0.52	15	0.61
Prefer N. A.	10	0.38	6	0.24	3	0.12

E, East; N, North; Prefer N. A., Prefer not to answer; Sub-Saharan A., Sub-Saharan Africa; S, South; W, West

Table S4. Religion or religious attitude participants identified with, and weekly participation in religious services.

Religion / Religious attitude	N	Percent	Regular	Occasional	Rare	Not involved	N/A
Agnosticism	464	17.47	0.22	0.65	8.06	71.68	19.39
Atheism	824	31.02	0.49	0.12	2.08	65.44	31.86
Buddhism	88	3.31	13.79	9.20	22.99	44.83	9.20
Catholicism	486	18.30	24.07	14.94	28.84	30.91	1.24
Hinduism	39	1.47	18.42	28.95	21.05	23.68	7.89
Islam	48	1.81	29.17	22.92	22.92	20.83	4.17
Judaism	90	3.39	13.48	17.98	34.83	33.71	0
Orthodox Christianity	50	1.88	4	18	44	34	0
Protestant/Evangelical	203	7.64	28.71	20.79	23.27	27.23	0
Traditional/Indigenous	8	0.30	37.5	0	12.5	50	0
Other	161	6.06	16.25	4.38	8.13	47.50	23.75
Prefer not to answer	195	7.34	2.76	2.76	9.94	44.75	39.78

Table S5. Political self-identification of the respondents

Political attitude	N	Percent
Very conservative	12	0.45
Conservative	152	5.73
Moderate	504	19
Liberal	1134	42.76
Very liberal	249	9.39
No identification	335	12.63
Other	66	2.49
Prefer not to answer	200	7.54

Table S6. Relevance of the Mind-Brain Problem, attitudes towards it and future of neuroscience

MBP important for:	Strongly agree	Agree	Neutral	Disagree	Strongly disagree
One's research	1013 (38.17)	746 (28.11)	423 (15.94)	257 (9.68)	215 (8.1)
Understanding humans	1124 (42.43)	868 (32.77)	492 (18.57)	114 (4.30)	51 (1.93)
Neuroscience	1347 (50.75)	831 (31.31)	328 (12.36)	86 (3.24)	62 (2.34)
**Attitude**	**Strongly agree**	**Agree**	**Neutral**	**Disagree**	**Strongly disagree**
Reductive physicalism	995 (37.5)	693 (26.12)	383 (14.44)	373 (14.06)	209 (7.88)
Dualism	452 (17.04)	521 (19.64)	455 (17.15)	494 (18.62)	731 (27.55)
Non-reductive physic.	393 (14.84)	744 (28.1)	385 (14.54)	513 (19.37)	613 (23.15)
Dual-aspect monism	417 (15.72)	669 (25.22)	469 (17.68)	484 (18.24)	614 (23.14)
Functionalism	291 (11.01)	626 (23.68)	773 (29.24)	503 (19.02)	451 (17.06)
**Proposition**	**Strongly agree**	**Agree**	**Neutral**	**Disagree**	**Strongly disagree**
Fully understand humans	424 (16.23)	984 (37.67)	542 (20.75)	435 (16.65)	227 (8.69)
Mind reading	219 (8.37)	784 (29.97)	635 (24.27)	656 (25.08)	322 (12.31)
Mental uploading	99 (3.78)	473 (18.07)	691 (26.4)	730 (27.89)	624 (23.84)
Reality is a simulation	370 (14.14)	799 (30.53)	631 (24.11)	418 (15.97)	399 (15.25)
Mental illness=brain dis.	387 (14.83)	824 (31.58)	507 (19.43)	607 (23.27)	284 (34.15)
Any mental process	331 (12.66)	962 (36.79)	540 (20.65)	552 (21.11)	230 (8.8)
Hard problem consc.	210 (8.05)	786 (30.13)	755 (28.94)	576 (22.08)	282 (10.81)

Level of agreement with the relevance of the mind-brain problem (MBP) (top), with different statements showing the most common attitudes towards the MBP (middle), and with some statements about the present and future of neuroscience. "Neutral" was shown as "neither agree nor disagree." Results are shown as the number of respondents with percentage in parentheses.

Table S7. Perceived vs actual percentage of agreement with each mind-brain problem position.

Attitude	Perceived agreement (%)	Actual agreement (%)
Reductive physicalism	74.39	63.62
Dualism	34.34	36.68
Non-reductive physic.	33.18	42.94
Dual-aspect monism	38.03	40.94
Functionalism	37.53	34.69

‘Actual agreement’ is the percentage of participants who voted 3 or 4 in the survey questions about the attitudes towards the mind-brain problem (questions 4 to 8). ‘Perceived agreement’ is the percentage of participants who considered each position frequent or very frequent (i.e., voted 3 or 4 in the final questions of the survey).

Table S8. Effects of sociodemographic variables on component 1 ("*neurooptimism*")

		Coefficient	S. E.	t value	p value	C.I. 95%	
Gender	Female	-0.058	0.046	-1.280	0.201	-0.148	0.031
(ref. male)	Other	-0.387	0.363	-1.065	0.287	-1.099	0.325
	Blank	0.317	0.202	1.570	0.117	-0.079	0.713
Academic backg.	Natural sci.	-0.004	0.059	-0.071	0.943	-0.119	0.111
(ref. Health Sc.)	Social sci.	-0.326	0.069	-4.701	0.000	-0.462	-0.190
	Eng. and Tech.	-0.082	0.113	-0.722	0.470	-0.304	0.140
	Humanities	-0.920	0.242	-3.806	0.000	-1.393	-0.446
	Other	-0.254	0.158	-1.604	0.109	-0.565	0.057
	Several	-0.103	0.068	-1.515	0.130	-0.236	0.030
Research area	Developmental	-0.095	0.135	-0.701	0.483	-0.360	0.170
(ref. Several)	Cognitive	-0.069	0.071	-0.971	0.332	-0.210	0.071
	Mol and Cell	0.186	0.076	2.442	0.015	0.037	0.335
	Genetics	-0.042	0.189	-0.221	0.825	-0.413	0.329
	Systems	0.115	0.088	1.314	0.189	-0.057	0.287
	Computational	-0.129	0.138	-0.935	0.350	-0.398	0.141
	Translational	0.063	0.107	0.589	0.556	-0.146	0.272
	Ethics	-0.227	0.370	-0.612	0.541	-0.953	0.500
	Philosophy	-0.260	0.238	-1.094	0.274	-0.727	0.206
	Clinical	-0.147	0.069	-2.136	0.033	-0.281	-0.012
	Other	-0.094	0.083	-1.135	0.257	-0.256	0.068
	Prefer not to answer	0.131	0.285	0.459	0.646	-0.428	0.690
World region lived	North America	0.038	0.051	0.747	0.455	-0.062	0.138
(ref. West. Europe)	Central Am. Caribbean	0.390	0.243	1.607	0.108	-0.086	0.866
	South America	0.170	0.103	1.643	0.100	-0.033	0.373
	Eastern Europe	0.282	0.097	2.899	0.004	0.091	0.473
	Northern Africa	0.285	0.696	0.410	0.682	-1.080	1.650
	Sub-Saharan Africa	-0.040	0.305	-0.130	0.897	-0.639	0.559
	East Asia	0.449	0.113	3.969	0.000	0.227	0.671
	South Asia	0.271	0.203	1.336	0.182	-0.127	0.670
	Central Asia	0.764	0.687	1.111	0.267	-0.584	2.112
	Middle East	0.282	0.163	1.733	0.083	-0.037	0.600
	Oceania	-0.044	0.114	-0.386	0.699	-0.267	0.179
	Other	0.405	0.270	1.502	0.133	-0.124	0.934
	Prefer not to answer	-0.336	0.686	-0.489	0.625	-1.682	1.010
Religion	Agnosticism	-0.277	0.061	-4.552	0.000	-0.396	-0.158
(ref. atheism)	Buddhism	-0.480	0.125	-3.837	0.000	-0.725	-0.235
	Catholicism	-0.134	0.064	-2.078	0.038	-0.260	-0.008
	Hinduism	-0.023	0.207	-0.111	0.911	-0.428	0.382
	Islam	0.208	0.173	1.201	0.230	-0.132	0.548
	Judaism	-0.372	0.118	-3.142	0.002	-0.604	-0.140
	Orthodox Christianity	-0.149	0.159	-0.940	0.348	-0.460	0.162
	Protestant/Evangelical	-0.483	0.085	-5.716	0.000	-0.649	-0.317
	Traditional/Indigenous	-0.583	0.375	-1.552	0.121	-1.319	0.154
	Other	-0.268	0.094	-2.835	0.005	-0.453	-0.082
	Prefer not to answer	-0.013	0.095	-0.138	0.890	-0.200	0.173
Politics	Very conservative	-0.237	0.323	-0.734	0.463	-0.871	0.397
(ref. liberal)	Conservative	-0.173	0.095	-1.813	0.070	-0.360	0.014
	Moderate	0.040	0.058	0.698	0.485	-0.073	0.154
	Very liberal	-0.088	0.074	-1.185	0.236	-0.234	0.058

		Coefficient	S. E.	t value	p value	C.I. 95%	
	No identification	0.029	0.070	0.411	0.681	-0.108	0.165
	Other	-0.113	0.140	-0.808	0.419	-0.388	0.162
	Prefer not to answer	0.077	0.092	0.834	0.404	-0.104	0.259
Age range	18-29	-0.301	0.166	-1.815	0.070	-0.626	0.024
(ref. 40-49 yr)	30-39	-0.137	0.067	-2.041	0.041	-0.268	-0.005
	50-59	-0.014	0.057	-0.244	0.807	-0.125	0.097
	60-69	0.005	0.063	0.073	0.942	-0.118	0.128
	70-79	-0.023	0.084	-0.272	0.786	-0.188	0.142
	80+	0.243	0.138	1.762	0.078	-0.027	0.513
Constant		0.243	0.073	3.314	0.001	0.099	0.387

Table S9. Effects of sociodemographic variables on component 2 (“*relevance of the mind-brain problem”*)

		Coefficient	S.E.	t value	p value	C.I. 95%	
Gender	Female	0.035	0.046	0.745	0.456	-0.057	0.126
(ref. male)	Other	-0.113	0.371	-0.305	0.761	-0.840	0.614
	Blank	-0.389	0.206	-1.888	0.059	-0.793	0.015
Academic backg.	Natural sci.	-0.111	0.060	-1.846	0.065	-0.229	0.007
(ref. Health Sc.)	Social sci.	0.121	0.071	1.710	0.087	-0.018	0.260
	Eng. and Tech.	-0.010	0.116	-0.089	0.929	-0.237	0.216
	Humanities	0.182	0.247	0.739	0.460	-0.301	0.666
	Other	0.344	0.162	2.126	0.034	0.027	0.661
	Several	0.080	0.069	1.147	0.252	-0.056	0.216
Research area	Developmental	-0.358	0.138	-2.593	0.010	-0.629	-0.087
(ref. Several)	Cognitive	0.123	0.073	1.679	0.093	-0.021	0.266
	Mol and Cell	-0.321	0.078	-4.127	0.000	-0.473	-0.168
	Genetics	-0.370	0.193	-1.914	0.056	-0.748	0.009
	Systems	-0.262	0.089	-2.932	0.003	-0.438	-0.087
	Computational	0.009	0.141	0.064	0.949	-0.267	0.285
	Translational	-0.044	0.109	-0.403	0.687	-0.258	0.170
	Ethics	-0.538	0.378	-1.423	0.155	-1.281	0.204
	Philosophy	-0.134	0.243	-0.552	0.581	-0.611	0.342
	Clinical	-0.153	0.070	-2.176	0.030	-0.290	-0.015
	Other	-0.391	0.085	-4.621	0.000	-0.557	-0.225
	Prefer not to answer	-0.342	0.291	-1.173	0.241	-0.912	0.229
World region lived	North America	-0.080	0.052	-1.536	0.125	-0.182	0.022
(ref. West. Europe)	Central Am. Caribbean	0.294	0.248	1.186	0.236	-0.192	0.780
	South America	0.206	0.106	1.951	0.051	-0.001	0.413
	Eastern Europe	0.126	0.099	1.264	0.207	-0.069	0.320
	Northern Africa	1.062	0.711	1.494	0.135	-0.332	2.456
	Sub-Saharan Africa	0.389	0.312	1.247	0.212	-0.223	1.001
	East Asia	-0.159	0.116	-1.378	0.168	-0.386	0.067
	South Asia	0.040	0.207	0.191	0.849	-0.367	0.446
	Central Asia	-0.956	0.702	-1.361	0.174	-2.332	0.421
	Middle East	0.140	0.166	0.842	0.400	-0.186	0.465
	Oceania	0.078	0.116	0.672	0.502	-0.150	0.306
	Other	-0.272	0.275	-0.986	0.324	-0.812	0.269
	Prefer not to answer	1.039	0.701	1.482	0.138	-0.336	2.414
Religion	Agnosticism	-0.097	0.062	-1.557	0.120	-0.219	0.025
(ref. atheism)	Buddhism	0.121	0.128	0.949	0.343	-0.129	0.372
	Catholicism	0.051	0.066	0.779	0.436	-0.078	0.180
	Hinduism	-0.001	0.211	-0.002	0.998	-0.414	0.413
	Islam	-0.415	0.177	-2.348	0.019	-0.762	-0.068
	Judaism	-0.156	0.121	-1.295	0.195	-0.393	0.080
	Orthodox Christianity	0.127	0.162	0.784	0.433	-0.191	0.445
	Protestant/Evangelical	-0.129	0.086	-1.492	0.136	-0.298	0.041
	Traditional/Indigenous	-0.757	0.383	-1.975	0.048	-1.509	-0.005
	Other	-0.082	0.096	-0.850	0.395	-0.271	0.107
	Prefer not to answer	-0.049	0.097	-0.504	0.614	-0.239	0.141
Politics	Very conservative	0.511	0.330	1.549	0.122	-0.136	1.159
(ref. liberal)	Conservative	-0.042	0.097	-0.429	0.668	-0.233	0.149
	Moderate	0.024	0.059	0.404	0.686	-0.092	0.140
	Very liberal	0.076	0.076	1.003	0.316	-0.073	0.226

		Coefficient	S.E.	t value	p value	C.I. 95%	
	No identification	0.021	0.071	0.289	0.773	-0.119	0.160
	Other	0.114	0.143	0.792	0.428	-0.167	0.395
	Prefer not to answer	-0.065	0.094	-0.692	0.489	-0.251	0.120
Age range	18-29	-0.068	0.169	-0.399	0.690	-0.400	0.265
(ref. 40-49 yr)	30-39	-0.010	0.068	-0.152	0.880	-0.144	0.124
	50-59	0.096	0.058	1.666	0.096	-0.017	0.209
	60-69	0.183	0.064	2.853	0.004	0.057	0.308
	70-79	0.247	0.086	2.878	0.004	0.079	0.416
	80+	0.177	0.141	1.257	0.209	-0.099	0.453
Constant		0.048	0.075	0.644	0.519	-0.099	0.195

Table S10. Effects of sociodemographic variables on component 3 ("*non-reductionism*")

		Coefficient	S. E.	t value	p value	C.I. 95%	
Gender	Female	0.246	0.044	5.635	0.000	0.160	0.331
(ref. male)	Other	-0.328	0.348	-0.943	0.346	-1.010	0.354
	Blank	-0.347	0.193	-1.794	0.073	-0.726	0.032
Academic backg.	Natural sci.	-0.221	0.056	-3.933	0.000	-0.332	-0.111
(ref. Health Sc.)	Social sci.	-0.134	0.066	-2.016	0.044	-0.264	-0.004
	Eng. and Tech.	0.011	0.108	0.105	0.916	-0.201	0.224
	Humanities	-0.205	0.231	-0.884	0.377	-0.659	0.249
	Other	-0.142	0.152	-0.933	0.351	-0.439	0.156
	Several	-0.062	0.065	-0.950	0.342	-0.189	0.066
Research area	Developmental	0.162	0.130	1.246	0.213	-0.093	0.416
(ref. Several)	Cognitive	-0.163	0.068	-2.379	0.017	-0.297	-0.029
	Mol and Cell	0.081	0.073	1.107	0.269	-0.062	0.224
	Genetics	0.018	0.181	0.098	0.922	-0.337	0.373
	Systems	-0.223	0.084	-2.658	0.008	-0.388	-0.058
	Computational	-0.047	0.132	-0.360	0.719	-0.306	0.211
	Translational	-0.024	0.102	-0.239	0.811	-0.225	0.176
	Ethics	0.519	0.355	1.462	0.144	-0.177	1.215
	Philosophy	0.018	0.228	0.078	0.937	-0.429	0.465
	Clinical	-0.054	0.066	-0.822	0.411	-0.183	0.075
	Other	0.079	0.079	1.000	0.317	-0.076	0.235
	Prefer not to answer	-0.091	0.273	-0.334	0.739	-0.627	0.444
World region lived	North America	-0.007	0.049	-0.148	0.882	-0.103	0.089
(ref. West. Europe)	Central Am. Caribbean	0.246	0.232	1.059	0.290	-0.210	0.702
	South America	0.316	0.099	3.187	0.001	0.121	0.510
	Eastern Europe	0.172	0.093	1.850	0.065	-0.010	0.355
	Northern Africa	-0.318	0.667	-0.476	0.634	-1.626	0.990
	Sub-Saharan Africa	0.228	0.293	0.779	0.436	-0.346	0.802
	East Asia	0.533	0.108	4.916	0.000	0.320	0.746
	South Asia	0.196	0.195	1.007	0.314	-0.186	0.578
	Central Asia	1.434	0.659	2.178	0.030	0.143	2.726
	Middle East	0.155	0.156	0.996	0.319	-0.150	0.460
	Oceania	0.136	0.109	1.248	0.212	-0.078	0.350
	Other	0.086	0.258	0.333	0.739	-0.421	0.593
	Prefer not to answer	1.161	0.658	1.765	0.078	-0.129	2.451
Religion	Agnosticism	0.334	0.058	5.728	0.000	0.220	0.448
(ref. atheism)	Buddhism	0.504	0.120	4.206	0.000	0.269	0.739
	Catholicism	0.653	0.062	10.595	0.000	0.532	0.774
	Hinduism	1.086	0.198	5.490	0.000	0.698	1.475
	Islam	1.030	0.166	6.206	0.000	0.705	1.355
	Judaism	0.138	0.113	1.221	0.222	-0.084	0.361
	Orthodox Christianity	0.578	0.152	3.803	0.000	0.280	0.876
	Protestant/Evangelical	0.516	0.081	6.370	0.000	0.357	0.675
	Traditional/Indigenous	0.595	0.360	1.655	0.098	-0.110	1.301
	Other	0.307	0.090	3.399	0.001	0.130	0.485
	Prefer not to answer	0.453	0.091	4.976	0.000	0.275	0.632
Politics	Very conservative	0.357	0.310	1.152	0.249	-0.250	0.964
(ref. liberal)	Conservative	0.259	0.091	2.832	0.005	0.080	0.438
	Moderate	0.148	0.056	2.658	0.008	0.039	0.257
	Very liberal	0.036	0.071	0.500	0.617	-0.104	0.176

		Coefficient	S. E.	t value	p value	C.I. 95%	
	No identification	0.280	0.067	4.195	0.000	0.149	0.411
	Other	-0.057	0.134	-0.422	0.673	-0.320	0.207
	Prefer not to answer	0.098	0.089	1.110	0.267	-0.075	0.272
Age range	18-29	0.200	0.159	1.256	0.209	-0.112	0.511
(ref. 40-49 yr)	30-39	0.097	0.064	1.518	0.129	-0.028	0.223
	50-59	0.006	0.054	0.103	0.918	-0.101	0.112
	60-69	0.146	0.060	2.430	0.015	0.028	0.264
	70-79	0.103	0.081	1.280	0.201	-0.055	0.261
	80+	0.264	0.132	1.999	0.046	0.005	0.523
Constant		-0.507	0.070	-7.204	0.000	-0.645	-0.369

Table S11. Effects of sociodemographic variables on component 4 ("*computational paradigm*")

		Coefficient	S. E.	t value	p value	C.I. 95%	
Gender	Female	-0.082	0.045	-1.812	0.070	-0.170	0.007
(ref. male)	Other	0.367	0.360	1.018	0.309	-0.340	1.074
	Blank	0.004	0.200	0.021	0.983	-0.389	0.397
Academic backg.	Natural sci.	0.008	0.058	0.134	0.893	-0.107	0.122
(ref. Health Sc.)	Social sci.	0.218	0.069	3.169	0.002	0.083	0.353
	Eng. and Tech.	0.130	0.112	1.160	0.246	-0.090	0.351
	Humanities	0.400	0.240	1.670	0.095	-0.070	0.871
	Other	0.255	0.157	1.623	0.105	-0.053	0.564
	Several	0.237	0.067	3.515	0.000	0.105	0.369
Research area	Developmental	-0.092	0.134	-0.687	0.492	-0.355	0.171
(ref. Several)	Cognitive	0.186	0.071	2.618	0.009	0.047	0.325
	Mol and Cell	-0.396	0.076	-5.242	0.000	-0.544	-0.248
	Genetics	-0.252	0.188	-1.341	0.180	-0.620	0.116
	Systems	0.049	0.087	0.561	0.575	-0.122	0.219
	Computational	0.407	0.137	2.983	0.003	0.139	0.675
	Translational	-0.277	0.106	-2.613	0.009	-0.484	-0.069
	Ethics	-0.902	0.368	-2.454	0.014	-1.623	-0.181
	Philosophy	-0.034	0.236	-0.143	0.887	-0.497	0.429
	Clinical	-0.251	0.068	-3.683	0.000	-0.385	-0.117
	Other	-0.272	0.082	-3.312	0.001	-0.433	-0.111
	Prefer not to answer	-0.599	0.283	-2.120	0.034	-1.154	-0.045
World region lived	North America	0.071	0.051	1.411	0.158	-0.028	0.171
(ref. West. Europe)	Central Am. Caribbean	-0.355	0.241	-1.475	0.140	-0.827	0.117
	South America	0.031	0.103	0.300	0.764	-0.170	0.232
	Eastern Europe	-0.007	0.096	-0.068	0.946	-0.196	0.183
	Northern Africa	0.422	0.691	0.611	0.541	-0.933	1.776
	Sub-Saharan Africa	0.348	0.303	1.148	0.251	-0.246	0.942
	East Asia	0.363	0.112	3.236	0.001	0.143	0.583
	South Asia	0.051	0.202	0.253	0.800	-0.344	0.446
	Central Asia	-0.235	0.682	-0.345	0.730	-1.573	1.102
	Middle East	0.004	0.161	0.027	0.978	-0.312	0.321
	Oceania	0.045	0.113	0.397	0.691	-0.176	0.266
	Other	-0.397	0.268	-1.482	0.138	-0.921	0.128
	Prefer not to answer	0.442	0.681	0.650	0.516	-0.893	1.778
Religion	Agnosticism	-0.007	0.060	-0.112	0.910	-0.125	0.112
(ref. atheism)	Buddhism	-0.093	0.124	-0.748	0.455	-0.336	0.151
	Catholicism	-0.029	0.064	-0.454	0.650	-0.154	0.096
	Hinduism	0.024	0.205	0.115	0.909	-0.378	0.425
	Islam	0.077	0.172	0.448	0.654	-0.260	0.414
	Judaism	0.111	0.117	0.942	0.346	-0.120	0.341
	Orthodox Christianity	-0.214	0.157	-1.361	0.174	-0.523	0.094
	Protestant/Evangelical	-0.065	0.084	-0.777	0.437	-0.230	0.099
	Traditional/Indigenous	0.305	0.373	0.819	0.413	-0.425	1.036
	Other	-0.018	0.094	-0.190	0.850	-0.201	0.166
	Prefer not to answer	0.011	0.094	0.112	0.911	-0.174	0.196
Politics	Very conservative	-0.706	0.321	-2.200	0.028	-1.335	-0.077
(ref. liberal)	Conservative	-0.167	0.095	-1.765	0.078	-0.353	0.019
	Moderate	-0.085	0.058	-1.478	0.140	-0.198	0.028
	Very liberal	-0.093	0.074	-1.264	0.206	-0.238	0.052

		Coefficient	S. E.	t value	p value	C.I. 95%	
	No identification	-0.175	0.069	-2.537	0.011	-0.311	-0.040
	Other	0.054	0.139	0.391	0.696	-0.219	0.327
	Prefer not to answer	-0.085	0.092	-0.931	0.352	-0.265	0.095
Age range	18-29	0.351	0.165	2.133	0.033	0.028	0.674
(ref. 40-49 yr)	30-39	0.129	0.066	1.946	0.052	-0.001	0.260
	50-59	-0.133	0.056	-2.369	0.018	-0.243	-0.023
	60-69	-0.255	0.062	-4.103	0.000	-0.377	-0.133
	70-79	-0.305	0.084	-3.654	0.000	-0.469	-0.141
	80+	-0.672	0.137	-4.913	0.000	-0.940	-0.404
Constant	Constant	0.162	0.073	2.219	0.027	0.019	0.304

Table S12. Effects of sociodemographic variables on component 5 (“*determinism*”)

		Coefficient	S. E.	t value	p value	C.I. 95%	
Gender	Female	-0.167	0.047	-3.583	0.000	-0.258	-0.075
(ref. male)	Other	0.121	0.371	0.325	0.745	-0.608	0.849
	Blank	0.198	0.206	0.961	0.336	-0.206	0.603
Academic backg.	Natural sci.	0.053	0.060	0.889	0.374	-0.064	0.171
(ref. Health Sc.)	Social sci.	0.082	0.071	1.154	0.249	-0.057	0.221
	Eng. and Tech.	-0.237	0.116	-2.047	0.041	-0.464	-0.010
	Humanities	-0.204	0.247	-0.827	0.408	-0.689	0.280
	Other	-0.054	0.162	-0.336	0.737	-0.372	0.263
	Several	0.128	0.069	1.840	0.066	-0.008	0.264
Research area	Developmental	0.047	0.138	0.338	0.736	-0.225	0.318
(ref. Several)	Cognitive	0.006	0.073	0.076	0.939	-0.138	0.149
	Mol and Cell	-0.102	0.078	-1.310	0.190	-0.255	0.051
	Genetics	0.190	0.193	0.984	0.325	-0.189	0.569
	Systems	0.071	0.090	0.795	0.427	-0.104	0.247
	Computational	0.046	0.141	0.327	0.743	-0.230	0.322
	Translational	0.023	0.109	0.211	0.833	-0.191	0.237
	Ethics	0.243	0.379	0.641	0.522	-0.500	0.986
	Philosophy	-0.240	0.243	-0.986	0.324	-0.717	0.237
	Clinical	0.055	0.070	0.787	0.431	-0.082	0.193
	Other	0.008	0.085	0.096	0.924	-0.158	0.174
	Prefer not to answer	0.068	0.291	0.232	0.817	-0.504	0.639
World region lived	North America	-0.085	0.052	-1.628	0.104	-0.187	0.017
(ref. West. Europe)	Central Am. Caribbean	0.348	0.248	1.404	0.160	-0.138	0.835
	South America	0.010	0.106	0.095	0.925	-0.197	0.217
	Eastern Europe	0.197	0.099	1.983	0.048	0.002	0.392
	Northern Africa	1.538	0.712	2.161	0.031	0.142	2.934
	Sub-Saharan Africa	-0.040	0.312	-0.129	0.898	-0.653	0.572
	East Asia	0.129	0.116	1.116	0.264	-0.098	0.356
	South Asia	0.298	0.208	1.435	0.151	-0.109	0.705
	Central Asia	0.268	0.703	0.381	0.703	-1.110	1.646
	Middle East	0.137	0.166	0.822	0.411	-0.189	0.463
	Oceania	-0.117	0.116	-1.008	0.313	-0.345	0.111
	Other	0.091	0.276	0.331	0.740	-0.449	0.632
	Prefer not to answer	0.172	0.702	0.245	0.807	-1.205	1.548
Religion	Agnosticism	-0.160	0.062	-2.574	0.010	-0.282	-0.038
(ref. atheism)	Buddhism	0.124	0.128	0.973	0.331	-0.126	0.375
	Catholicism	-0.433	0.066	-6.582	0.000	-0.562	-0.304
	Hinduism	0.143	0.211	0.676	0.499	-0.271	0.557
	Islam	-0.109	0.177	-0.616	0.538	-0.456	0.238
	Judaism	-0.192	0.121	-1.585	0.113	-0.429	0.045
	Orthodox Christianity	-0.227	0.162	-1.398	0.162	-0.545	0.091
	Protestant/Evangelical	-0.319	0.086	-3.696	0.000	-0.489	-0.150
	Traditional/Indigenous	0.268	0.384	0.698	0.485	-0.485	1.021
	Other	-0.182	0.097	-1.885	0.060	-0.371	0.007
	Prefer not to answer	-0.295	0.097	-3.033	0.002	-0.485	-0.104
Politics	Very conservative	0.360	0.331	1.088	0.277	-0.289	1.008
(ref. liberal)	Conservative	-0.230	0.098	-2.363	0.018	-0.422	-0.039
	Moderate	0.018	0.059	0.305	0.761	-0.098	0.134
	Very liberal	-0.089	0.076	-1.163	0.245	-0.238	0.061
	No identification	0.180	0.071	2.524	0.012	0.040	0.319

		Coefficient	S. E.	t value	p value	C.I. 95%	
	Other	-0.066	0.143	-0.463	0.643	-0.348	0.215
	Prefer not to answer	0.138	0.095	1.460	0.145	-0.047	0.323
Age range	18-29	-0.041	0.170	-0.244	0.807	-0.374	0.291
(ref. 40-49 yr)	30-39	0.135	0.068	1.974	0.049	0.001	0.269
	50-59	0.069	0.058	1.188	0.235	-0.045	0.182
	60-69	-0.074	0.064	-1.150	0.250	-0.199	0.052
	70-79	-0.008	0.086	-0.092	0.927	-0.177	0.161
	80+	-0.124	0.141	-0.882	0.378	-0.401	0.152
Constant		0.148	0.075	1.973	0.049	0.001	0.295

SI References

1. J. Fischer, R. Kane, D. Pereboom, M. Vargas, *Four Views on Free Will* (Blackwell Publishing, 2007).
2. W. Schultz, Neuroessentialism: Theoretical and Clinical Considerations. *J. Humanist. Psychol.* **58**, 607–639 (2018).
3. A. Singh, *et al.*, Transfer learning via distributed brain recordings enables reliable speech decoding. *Nat. Commun.* **16**, 8749 (2025).
4. G. Piccinini, "The Myth of Mind Uploading" in *The Mind-Technology Problem*, (Springer, 2021), pp. 125–144.
5. T. Metzinger, *Being no one: The self-model theory of subjectivity* (Cambridge University Press, 2003).
6. K. Friston, Prediction, perception and agency. *Int. J. Psychophysiol.* **83**, 248–252 (2012).
7. J. Parnas, S. Gallagher, Phenomenology and the interpretation of psychopathological experience. *Re-Visioning Psychiatry*, 65–80 (2015).
8. T. Fuchs, Why Does Mental Illness Exist ? Reflections on Human Vulnerability (2023).
9. G. Graham, *The disordered mind. An introduction to philosophy of mind and mental illness*, 2nd Ed. (Routledge, 2013).
10. N. Fabiano, *et al.*, The imaginary divide between mental and "physical" health: Dismantling dualism and reductionism to address a monumental mistake in medicine. *JCPP Adv.*, e70123 (2026).
